# High uptake of sympagic organic matter by benthos on an Arctic outflow shelf

**DOI:** 10.1371/journal.pone.0308562

**Published:** 2024-08-07

**Authors:** Ivan J. Cautain, Kim S. Last, Bodil A. Bluhm, Paul E. Renaud, David McKee, Bhavani E. Narayanaswamy

**Affiliations:** 1 Scottish Association for Marine Science, Oban, Scotland; 2 Department of Arctic and Marine Biology, UiT—The Arctic University of Norway, Tromsø, Norway; 3 Akvaplan-niva, Tromsø, Norway; 4 Arctic Biology Department, University Centre in Svalbard, Longyearbyen, Norway; 5 Physics Department, University of Strathclyde, Glasgow, Scotland; University of Maryland Center for Environmental Science, UNITED STATES OF AMERICA

## Abstract

On Arctic shelves, benthic food-webs are tightly linked to overlying primary production. In the seasonal ice zone, sympagic (ice-associated) primary production can be a major source of carbon for the benthos on productive inflow shelves. However, the role of sympagic organic matter is less well-understood in food webs of heavily ice-covered, less- productive outflow shelves, such as the northeast Greenland shelf. Highly branched isoprenoid biomarkers (HBIs) were used to track the relative distribution of sympagic and pelagic organic matter in the water column, sediments, and benthic fauna of the northeast Greenland shelf and fjords. Low pelagic HBI presence throughout the study area indicated a generally low production by pelagic diatoms (at the time of sampling). This was reflected in the benthos, as ~90% of their assimilated carbon was estimated to come from sympagic sources, indicating a benthic food-web highly reliant on sympagic production. This reliance was higher in coastal areas than on the open shelf, where the potentially higher pelagic productivity and shallower water on banks likely increased contributions of pelagic organic matter. As declining ice cover and reduced production of fast-sinking ice algae projected for Arctic shelves will likely result in weaker coupling between ice algae and the benthos, with possible consequences for future benthic-community structure and function.

## 1. Introduction

Sea ice is a major feature of the Arctic Ocean and is declining rapidly in many regions due to a warming climate [1]. Sea ice plays an important role in primary production by reducing the amount of light entering the marine system, affecting the timing of the spring bloom, and providing a habitat for ice-algae communities [2]. Ice-algae generally reach maximum biomass in spring, preceding the pelagic phytoplankton bloom which occurs once irradiance reaches suitable levels [3, 4]. Arctic phytoplankton bloom succession is dictated primarily by seeding (from sea ice), irradiance and nutrient concentrations [4, 5]. Typically, Arctic phytoplankton communities shift from a dominance of pennate diatoms (usually released from sea ice), to chain-forming centric diatoms, until they deplete nutrient levels in the upper water column and are succeeded by smaller-celled flagellates [5, 6].Arctic food-webs in ice-covered areas are therefore supported by both sympagic (ice-associated) and pelagic organic matter (OM), although in near-shore areas, macroalgae, microphytobenthos and terrestrial OM can also be important carbon sources [7–9]. Generally, the contribution of sympagic production to annual primary production is higher where there is more sea ice, largely due to the reduction in phytoplankton productivity [10]. As sea-ice extent and duration is rapidly decreasing due to climate change, pelagic production (and total annual production) is predicted to increase in many areas, at least in the short term, leading to changing contributions of sympagic and pelagic OM to Arctic food-webs [11, 12].

The benthos is a key component of Arctic ecosystems, as benthic communities play an important role in carbon flows and organic matter cycling [13–15]. On Arctic shelves, where there is generally tight sympagic-pelagic-benthic coupling, the benthos is the endpoint for much of the OM produced in the euphotic zone [15, 16]. Benthic food-webs largely reflect patterns in primary productivity, relying more on sympagic OM where there is high annual sea-ice presence [17, 18]. Although ice algae usually contribute to less than half of the total annual primary production [19–21], they are thought to be an important food source for the benthos and represent a significant early input of labile food due to the timing and speed of the sedimentation of ice algal diatoms [13]. Timing of food input in the highly seasonal Arctic can be critical for the reproduction and development of invertebrates, with high quality food being especially important for juvenile stages [22, 23]. Feeding experiments suggest that sympagic OM may be of high nutritional value and a preferred source of food for certain benthic invertebrates [24] due to its high essential fatty-acid content [25].

Estimating the contribution of sympagic and pelagic OM to Arctic benthic food-webs is key to understanding current carbon sourcing and predicting how changes in primary production may affect these communities in the future. Biomarkers, such as stable isotope ratios and fatty acids, are commonly used to track the flow of OM through Arctic food-webs (e.g., [26–28]). More recently, highly branched isoprenoids (HBIs) have been shown to effectively discriminate between sympagic and pelagic carbon sources. Certain 25-carbon HBIs are only produced either by species of sympagic diatoms (such as IP_25_ and HBI II–also named IPSO_25_) or by pelagic diatoms (such as HBI III) [29, 30]. As these molecules are transferred through food webs, the ratio of sympagic-to-pelagic HBIs in the tissue of organisms is used to estimate the proportion of assimilated OM that originated from sympagic or pelagic production [31].

HBI analyses on benthic fauna have improved our understanding of the role of sympagic and pelagic OM in benthic food-webs. On ice-covered Arctic shelves, sympagic OM is estimated to represent more than half of the carbon assimilated by megabenthos in the summer, but less than a third in the winter [17, 18, 32]. On inflow shelves, the contribution of sympagic OM from ice algae to benthic diets is strongly linked to annual sea-ice duration e.g. in the Bering and Chukchi Seas [17] and Barents Sea [18]. In Baffin Bay, a deep region on an outflow shelf, the contribution of sympagic OM to benthic diets was linked to sea-ice concentration [32]. Notably, these three regions are all relatively productive Arctic shelves, with a clear seasonality between the ice-free and ice-covered periods [33]. How the utilisation of sympagic and pelagic carbon varies on less productive Arctic shelves has not yet been explored but is of relevance in better understanding pan-Arctic variability in carbon transfer.

The northeast Greenland shelf is characterised by an outflow of sea ice and cold water (<0⁰C) from the Arctic basin, long fjords with marine-terminating glaciers [34] and ~10-month annual ice cover [35]. This results in low light and temperature conditions, which, combined with low nitrate concentrations, means low primary productivity over most of this shelf [36]. Net annual community primary production on the Greenland shelf is estimated at ~15 g C m^-2^, compared to the 60–100 g C m^-2^ (ice-covered Barents Sea) and ~70–100 g C m^-2^ (Bering and southern Chukchi Seas) of the more productive inflow shelves of the Arctic [33, 37]. This limited food input is reflected in a generally low abundance and biomass of benthos in fjords and on the shelf in northeast Greenland [38, 39]. There are however hotspots of high abundance and biomass associated with shallow banks and the Northeast Water polynya (78–81°N), which are areas of comparatively high pelagic primary production [39–41]. However, it is still largely unknown what roles sympagic and pelagic organic matter play in benthic food-webs outside of these hotspots of primary productivity [42].

The northeast Greenland shelf remains relatively understudied due to its remote location, extensive ice cover, and currently low economic value from natural resources. As a particularly cold Arctic shelf, it provides an interesting contrast to the well-studied, warm-water inflow shelves. This study provides an assessment of the distribution and relative assimilation of sympagic and pelagic HBIs and OM on the shelf and fjords of northeast Greenland, in the water column, sediments, and in benthic fauna. As the coastal areas and shelf also have different environmental conditions e.g., more fast ice and terrestrial run-off near the coast, and more drift ice, mixing and nutrients on the shelf [43, 44], fjord stations are expected to have higher signals of sympagic carbon throughout the food web since fast ice has been linked to higher sympagic OM export than drift ice [45, 46].

## 2. Methods

### 2.1 Sampling

**Fig 1:**
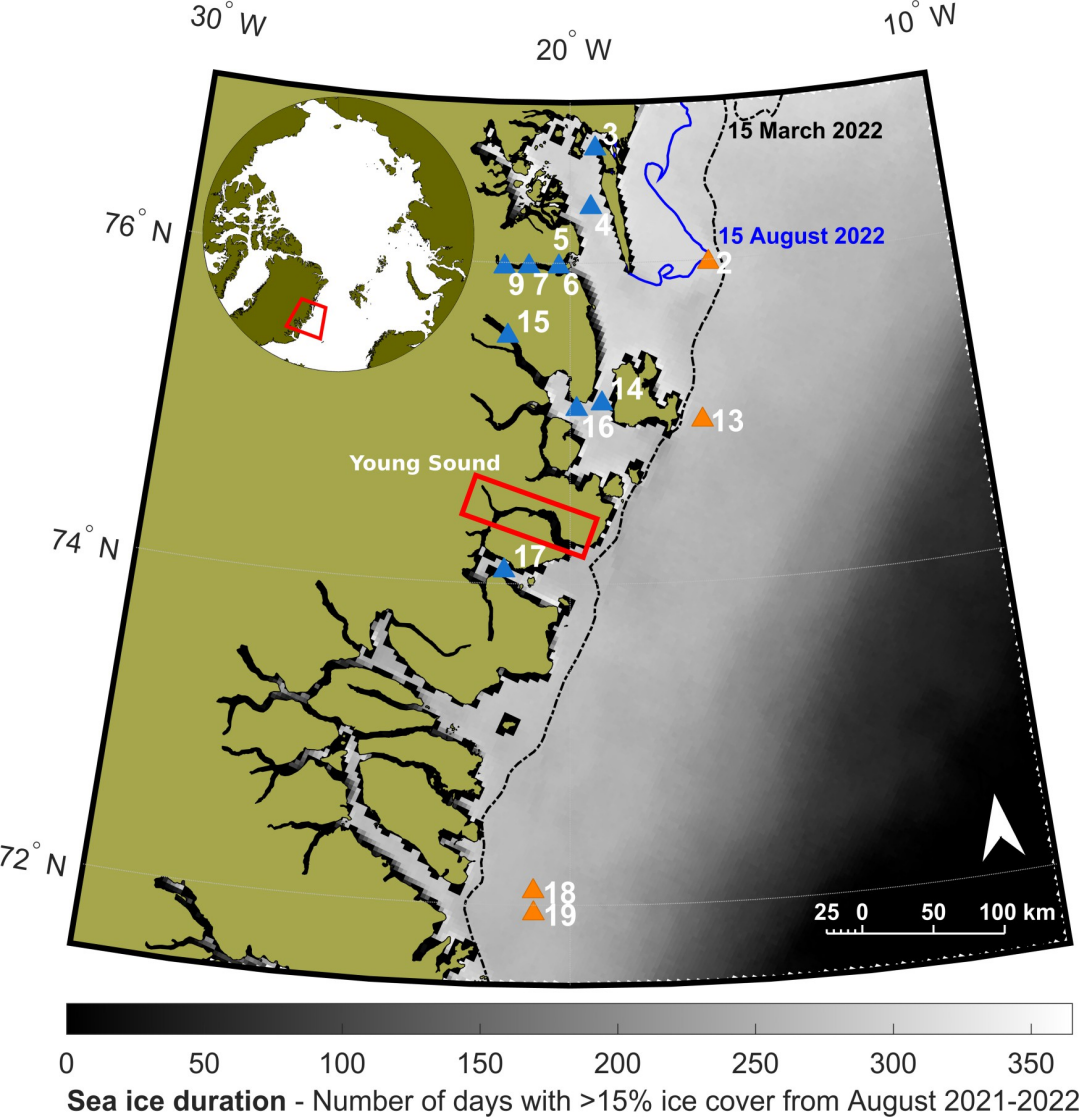
Map of study area showing sea ice duration. Sampling stations in northeast Greenland, with sea ice duration from August 2021 to August 2022. Colours represent different environments: blue–coastal, orange–shelf. Sea ice duration data are from gridded (resolution 3.125 km) satellite data downloaded from https://seaice.uni-bremen.de/sea-ice-concentration/ [48]. Lines show the extent of fast ice on the 15^th^ of March (dotted black) and 15^th^ of August 2022 (blue), obtained from the MET Norway Ice Service at https://cryo.met.no/en/latest-ice-charts. Coastline data are from Natural Earth [49].

Samples were collected at 14 stations on the northeast Greenland shelf and inside fjords, from the 28^th^ August to the 5^th^ September 2022, aboard the R/V *Kronprins Haakon* (Fig 1), during the TUNU VIII expedition under the KNNO Expedition Permit C-22-690 given by the Government of Greenland. Particulate organic matter (POM) from the water column, surface sediment, and benthic faunal samples were collected (see Table 1). Water for POM samples was collected with Niskin bottles at the chlorophyll *a* maximum and filtered (5.5–12 litres per sample) on pre-combusted GF/F filters. Sediment samples were collected with box cores: overlying water was first siphoned off, and 3–4 scoops of the top ca. 1 cm of sediment were collected with a spoon. Fauna were collected with Campelen and Agassiz trawls, and identified to the lowest practical taxonomic level onboard the ship. Where required to target specific tissues, dissections were also carried out onboard the ship: refer to raw data table for further details on tissues analysed (S1 Table, [47]). All samples were wrapped in aluminium foil and frozen at -20°C. All samples were oven-dried at 60°C onboard the ship. POM filters were dried for 2 h, sediment samples for 24 h, and fauna for 8–36 h, until fully dry.

**Table 1:** Sampling information for each station in the study in northeast Greenland.

Station	Latitude (°N)	Longitude (°W)	Depth (m)	SID (d y^-1^)	POM (depth)	Sediment	Fauna
2	75.982	16.397	70	308	X (32)		X
3	76.713	19.315	226	294	X (21)	X	X
4	76.348	19.450	411	300		X	
5	75.980	20.295	447	311*	X (34)		X
6	75.982	20.287	444	311*		X	
7	75.978	21.072	371	311*		X	
9	75.974	21.706	231	311*		X	X
13	75.003	16.767	342	274	X (43)	X	
14	75.125	19.216	82	289			X
15	75.543	21.576	563	287*	X (38)	X	X
16	75.091	19.841	343	295	X (28)	X	X
17	74.081	21.524	416	279	X (30)	X	X
18	72.086	20.759	210	261	X (45)		X
19	71.949	20.750	467	263		X	

SID is sea ice duration: asterisks denote stations where sea ice duration had to be calculated at the mouth of (rather than inside) the fjord due to low satellite resolution. Particulate organic matter (POM), Sediment and Fauna columns indicate whether samples of that type were collected at a given station (X–sample collected, blank–sample not collected). The number in parentheses in the POM column indicates the depth at which water was collected, in metres.

### 2.2 HBI extraction

All faunal samples were homogenised with a mortar and pestle prior to lipid extraction. HBI extractions were conducted according to previous studies [50], with an additional step for sediment samples. Elemental sulphur in sediments can interfere with the signal of HBI III and was therefore removed, as in [51]. After non-saponifiable lipids (NSL) were extracted, they were resuspended in hexane (2 ml). A tetrabutylammonium reagent (1 ml) and 2-propanol (2 ml) were added, and the sample vortexed for 1 min. Then, MilliQ water (3 ml) was added, and the sample vortexed for 1 min, before being centrifuged (2500 rpm; 2 min). The supernatant was transferred to a new vial. Hexane (2 ml) addition, vortex mixing, and centrifugation followed by supernatant extraction was carried out twice more, for a total of three times. The extracted supernatant was dried under N_2_ and resuspended in hexane (2 ml). Open column chromatography was then carried out as in previous studies (detail in [50]).

### 2.3 HBI analysis

Extracted HBIs were analysed in a Shimadzu QP2020 GC-MS with a 30m Rxi-5Sil column (0.25 mm i.d., 0.25 μm film), with operation parameters set according to [52]. Selective ion monitoring mode was used to target the ions characterising the three HBIs of interest: mass to charge ratios (*m/z*) for the sea ice algal markers of 350.3 for IP_25_ and 348.3 for HBI II, and 346.3 for the pelagic algal marker HBI III. The spectral intensities of these ions were then used to calculate the H-Print (Eq 1; [29]), where low values (<50%) indicate more sympagic HBIs, and high values (>50%) indicate more pelagic HBIs. For faunal samples, the H-Print was then used to calculate the proportion of assimilated OM from sympagic sources (known as % iPOC for “ice particulate organic carbon” in other studies), using an equation determined in a feeding experiment (Eq 2; [31]). Because this equation was determined in a feeding experiment, it was not used to estimate proportion of sympagic OM in POM or sediments, and they are therefore reported with their H-Print values.


$$H‐Print\;(\%)=\frac{HBI\;III}{\left({IP}_{25}+HBI\;II+HBI\;III\right)}\times 100$$ Eq 1

Sympagicorganicmatter%(%iPOC)=101.8−1.02×H‐Print Eq 2

### 2.4. Environmental parameters

Stations were grouped according to their habitat, as coastal or shelf stations. Coastal stations were those near the coast (including within fjords), with a stronger influence of landfast ice, freshwater and terrestrial run-off. Shelf stations were those found further offshore, with no fast ice cover for Stations 13, 18, and 19 (Fig 1), less freshwater influence, and a stronger input of Atlantic water. Sea-ice duration (SID) was calculated as [18], using gridded (resolution 3.125 km) satellite data downloaded from https://seaice.uni-bremen.de/sea-ice-concentration [48]. Due to cloud cover and low resolution in these narrow fjords, satellite resolution was not high enough to get reliable sea ice data for fjord stations (Fig 1). In these cases, SID was calculated at the mouth of the fjord, where measurements were more reliable–stations were 15–60 km from the point of SID estimate (Table 1). Once sea ice starts breaking up in spring, fjords can become ice-free within days to two weeks [53]. Due to the long SID present throughout the study area, this margin of error was not expected to cause differences in SID that would have a noticeable effect.

### 2.5. Statistical analyses

Statistical analyses were conducted in R 4.3.0 [54]. Only single replicates per station were taken for POM and sediment samples, so no statistical analyses were conducted on these data. A one-way ANOVA was used to determine if there were any significant differences in means of faunal H-Print values among stations, and a post-hoc Tukey HSD used to find what those differences were. A t-test was also conducted to compare the means of H-Print values in organisms between coastal and shelf stations. Linear models were used to explore the effects of SID and habitat on the proportion of sympagic carbon assimilated in benthos. Two models were made: a linear regression between SID and proportion of sympagic carbon in benthos, and a multiple linear regression with the added interaction of habitat type. Model fits were compared, and the better fitting model was used for graphing and interpretation.

## 3. Results

Overall, eight POM samples, ten sediment samples, and 281 faunal samples were collected from a total of 14 stations. One replicate was collected for POM and sediment samples, whereas 11 to 56 faunal samples were collected at stations where they were sampled (Table 2). This represented 47 taxa, of which 33 had more than one sample (S1 Fig). All three types of samples were collected at Stations (S) S3, S15, S16, and S17, although S5 (POM, fauna) and S6 (sediment), and S18 (POM, fauna) and S19 (sediment) were geographically close and had complementary samples.

**Table 2:** Distribution of target highly branched isoprenoids (HBIs) in northeast Greenland.

				*H-print*
Station	*n (l)*	IP_25_ (%)	II (%)	III (%)
*POM*				
2	1 (5.5)	-	-	-
3	1 (6.5)	-	-	-
5	1 (7.8)	-	-	-
13	1 (12)	-	-	-
15	1 (8.9)	-	-	-
16	1 (12)	-	-	-
17	1 (12)	-	-	-
18	1 (11.9)	-	-	-
*Sediment*				
3	1	53.5	46.5	-
4	1	48.8	49.3	1.9
6	1	46.5	53.5	-
7	1	48.1	51.9	-
9	1	38.0	58.7	3.3
13	1	27.7	65.9	6.4
15	1	40.2	59.8	-
16	1	41.7	54.4	3.9
17	1	48.3	51.7	-
19	1	26.5	66.8	6.7
*Fauna*				
2	45	24.4±2.5	52.1±6.0	23.5±8.3
3	33	40.4±3.6	57.2±3.2	2.5±1.1
5	31	34.3±4.1	59.7±3.1	6.0±3.2
9	24	33.5±4.7	61.5±6.7	4.9±3.0
14	19	27.1±2.2	57.4±5.9	15.5±5.8
15	11	29.2±1.8	62.7±2.11	8.1±2.3
16	30	31.2±3.4	55.8±4.2	13.0±5.9
17	32	33.4±3.1	58.3±4.0	8.3±2.4
18	56	24.2±4.5	62.0±5.3	13.8±5.2

HBIs in particulate organic matter (POM), sediment, and faunal samples at all stations sampled. Individual HBIs are shown as a proportion of the total HBIs of interest detected. Hyphens (-) indicate a lack of detection. *n* is the sample size, with the number in brackets indicating volume of water filtered for POM samples (in litres). IP_25_ and HBI II are sympagic HBIs, and HBI III is pelagic. The proportion of HBI III is equal to the H-print. For each faunal sample, the mean ± standard deviation of the HBI concentration is presented.

### 3.1. Distribution of HBIs

**Fig 2:**
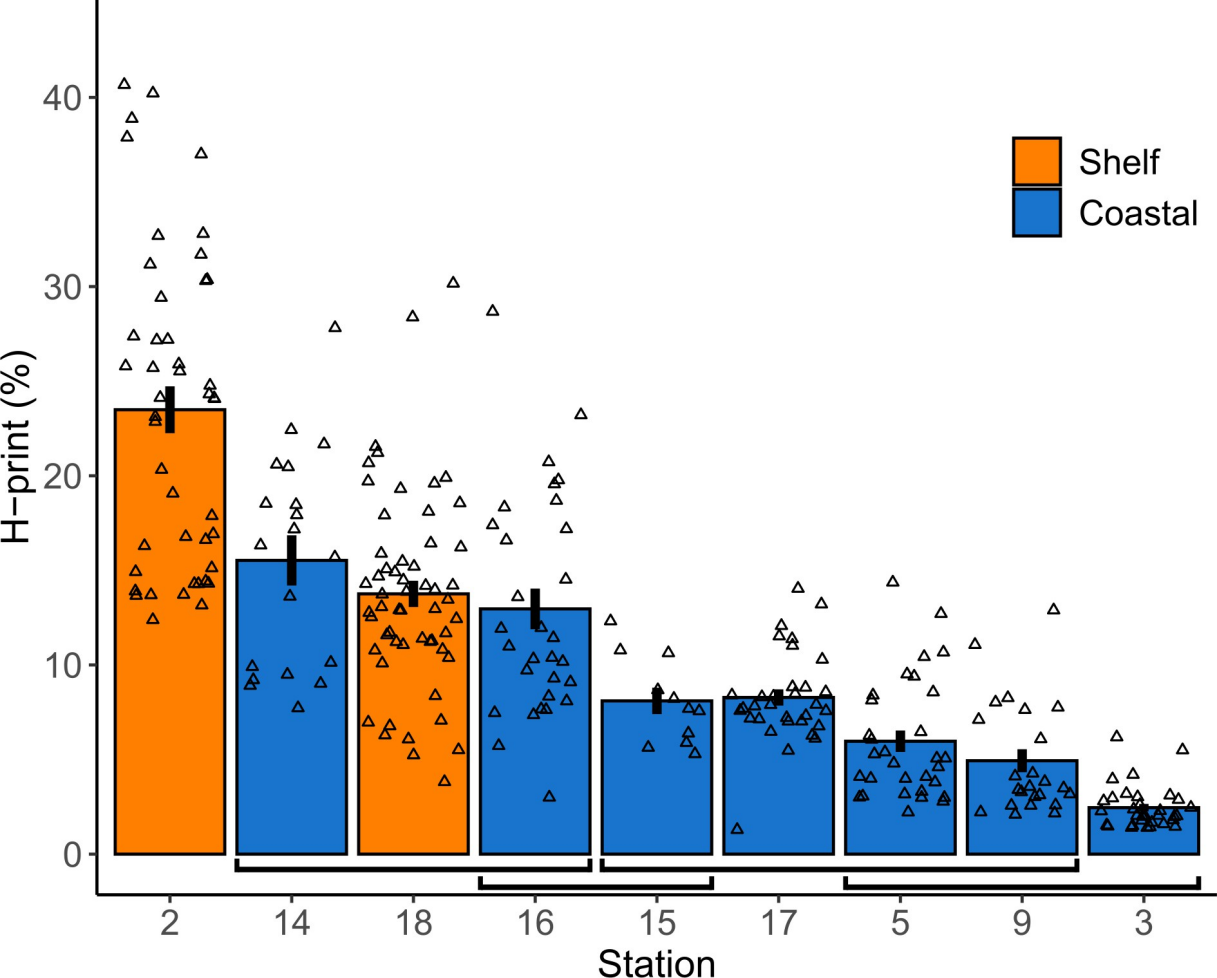
H-Print of benthos at each station. Mean H-Print of fauna at each station on the Northeast Greenland shelf. Black lines represent standard error, and open triangles represent individual data points. Stations are coloured according to habitat: orange are shelf stations; blue are coastal stations. Square brackets below the bars group statistically similar station means, determined by a post-hoc Tukey test.

No HBIs were detected in any of the POM samples, but all three target HBIs were detected in zoobenthos. While sympagic HBIs were detected in all sediment samples, the pelagic HBI III was only detected in half of the sediment samples (Table 2). This meant that the H-Print could not be calculated for five sediment samples. Sediment H-Prints averaged 4.0±2.5% (mean ± standard deviation), with the highest values (6.7% and 6.4%) at shelf stations, and the minimum (1.9%) at the coastal S4. H-Prints in fauna were higher, with an overall sample average of 11.6±8%. The highest average (23.5±8.3%) was at shelf S2, and the lowest (2.5±1.1%) at coastal S3 (Table 2). There were significant differences in the H-Print between stations (ANOVA, F(8, 272) = 60.36, p<0.001, Table 3). A post-hoc Tukey HSD showed that the two shelf stations with faunal samples had statistically higher mean H-Prints than most coastal stations (Fig 2; S2 Table): coastal S2 had a mean different from all other stations (including S18, p<0.001 in all cases), whereas shelf S18 had a mean that was only similar to coastal S14 and S16. Stations S14 and S16 were not significantly different from each other, but both had means higher than all other coastal stations (except S15 and S16, p = 0.1398; Fig 2). Most other coastal stations were statistically similar, but S3 had a lower mean H-Print than S15 and S17 (p = 0.039 and p<0.001, respectively).

**Table 3:** ANOVA results for benthic invertebrate H-Print.

	d.f	Sum Sq.	Mean Sq.	F-value	P-value
*Among stations*	8	12266	1533	60.36	**<0.001**
*Within stations*	272	6909	25		
*Total*	281	19175			

Results of one-way ANOVA comparing mean H-print of benthic invertebrates between stations from northeast Greenland. d.f.–Degrees of freedom; Sum Sq.—Sum of squares; Mean Sq.–Mean square.

### 3.2. Sympagic OM assimilation by fauna

**Fig 3:**
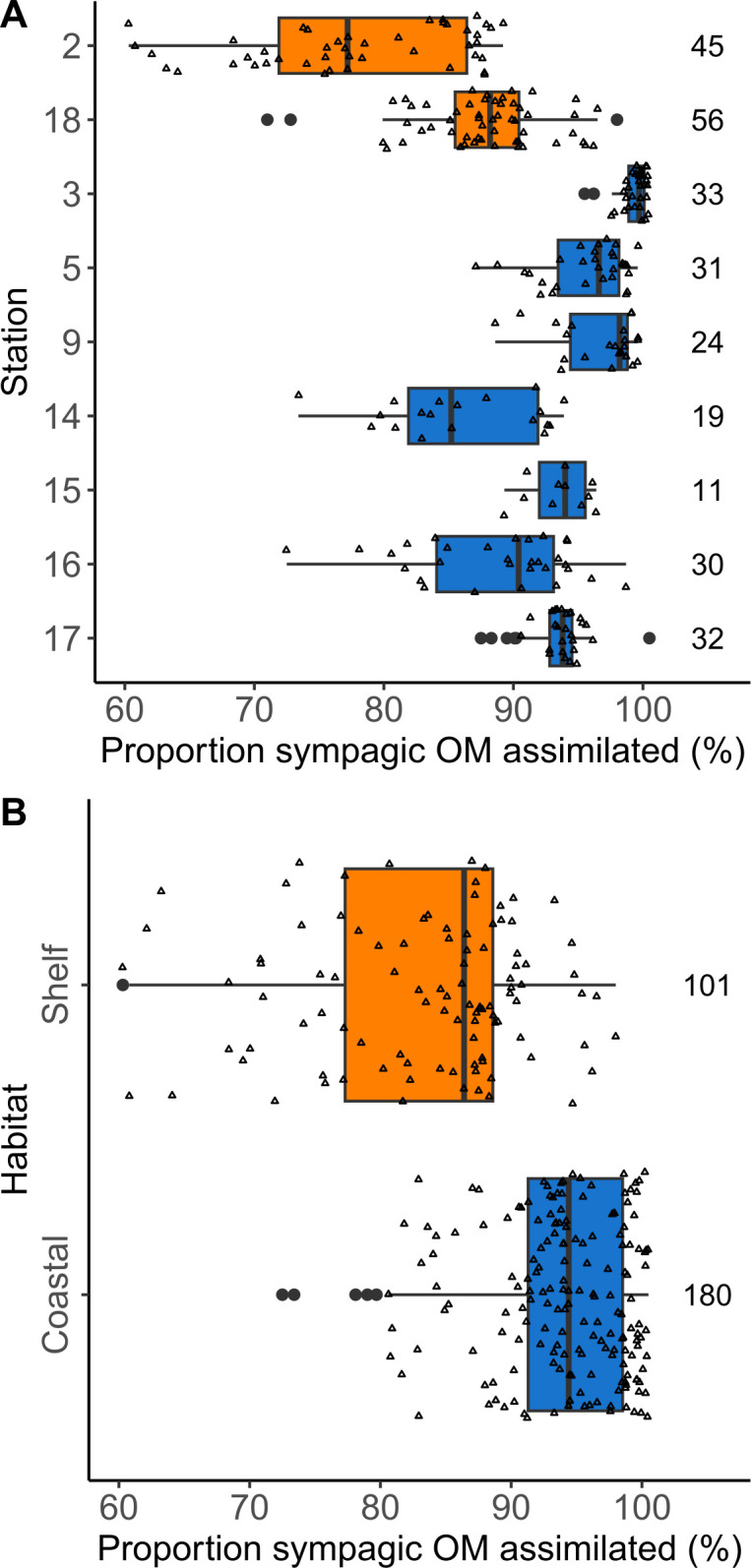
Proportion of sympagic organic matter in benthos. Estimates of the proportion of sympagic OM (iPOC %) assimilated in benthic invertebrates (a) at each station and (b) by habitat type, coloured according to habitat. Open triangles are individual data points, filled black circles are outliers. Boxes show the interquartile range. The vertical black line in each box is the median. Horizontal black lines include data points within 1.5 times the interquartile range of the lower and upper quartile. Numbers on the right are sample size. Note the restricted *x*-axis range.

H-Print calculations indicated that 90.0±8.4% of assimilated OM in all benthic fauna samples originated from sympagic production. The lowest estimated proportion of sympagic OM assimilated in a sample was 68.4% (the decapod *Sabinea septemcarinata* at S2), whereas three quarters of the samples had values of 85% or more. The lowest means by station were estimated for the shelf stations (S2: 77.8±8.5% and S18: 87.8±5.3%) and the coastal S14 (86.0±5.9%), whereas the highest mean was at coastal S3 (99±1.2%) (Fig 3). The ANOVA and Tukey results were very similar to the H-Print results, as these estimates are directly based on the H-Print (Eq 2; S3 and S4 Tables). A t-test looking at the effect of habitat on sympagic OM assimilation found a significant difference between shelf (83.3±8.5%) and coastal (93.7±5.7%) stations (df: 152; p-value<0.001).

**Fig 4:**
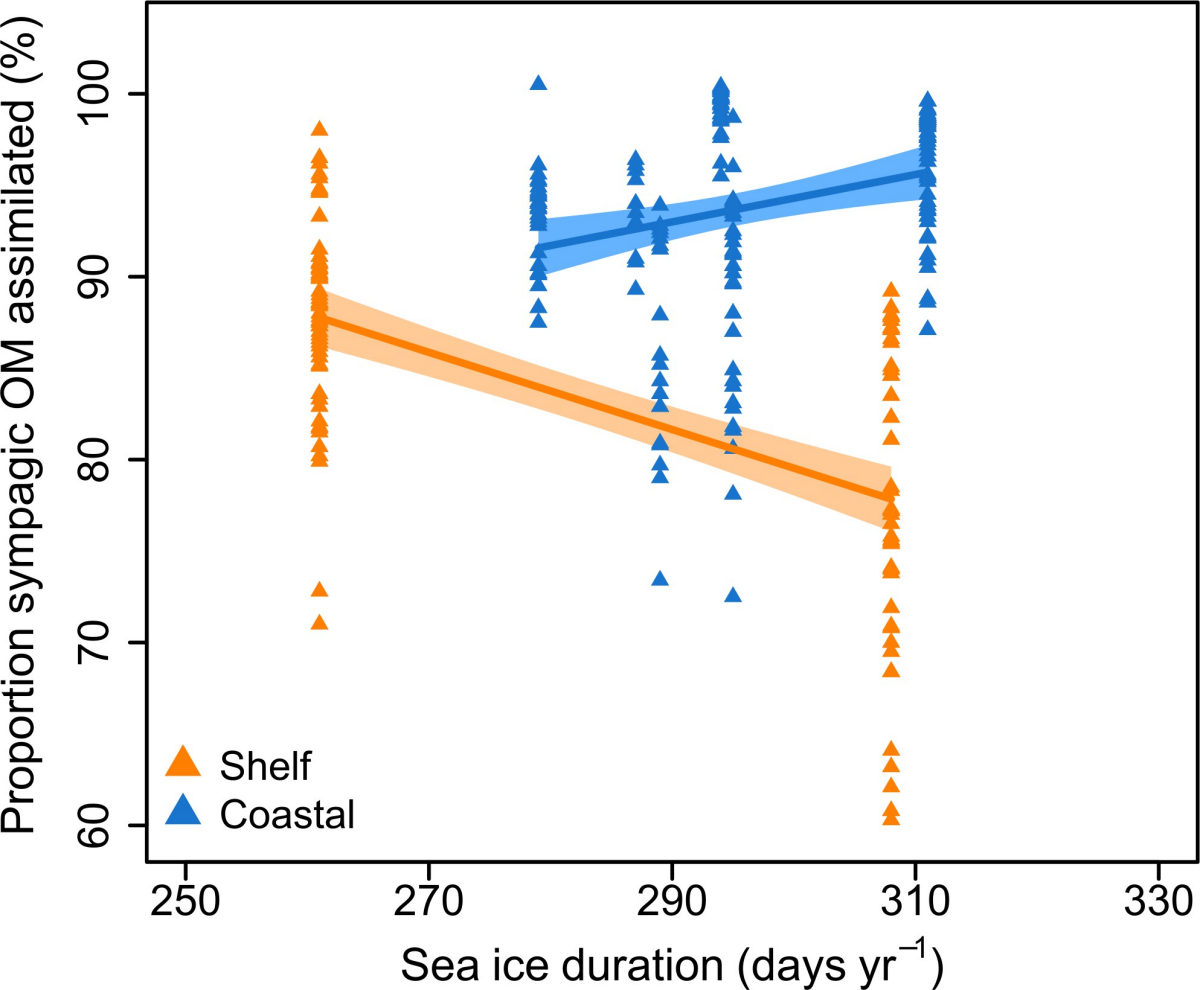
Relationship between sea ice duration and sympagic organic matter in benthos. Proportion of sympagic OM assimilated (iPOC %) in benthic invertebrates from northeast Greenland based on the H-Print approach against sea ice duration at each station, separated by habitat type: shelf stations are in orange, coastal stations are in blue. Lines represent a linear model for each habitat, with associated 95% confidence intervals. Note the restricted *x* and *y* axes ranges.

SID did not have a significant effect on the proportion of sympagic OM assimilated by fauna (Table 4): very little of the variation was explained by the simpler linear model (R^2^ = -0.003). The model with habitat (shelf/coastal) as an interacting factor fit the data better (R^2^ = 0.488) and indicated a significant effect on the slope (Fig 4; Table 4). However, the relationship between SID and proportion of sympagic OM assimilated remained non-significant (p-value = 0.699).

**Table 4:** Regression model diagnostics.

	R^2^	Parameters	d.f.	Sum Sq.	Mean Sq.	F-value	P-value
*Model 1*	-0.003	SID	1	5.4	5.4	0.076	0.783
			279	19939.1	71.4		
*Model 2*	0.488	SID	1	5.4	5.4	0.149	0.699
		Habitat	1	7922.2	7922.2	217.260	**<0.001**
		SID:Habitat	1	1916.4	1916.4	52.555	**<0.001**
			277	10100.6	36.5		
*Comparison*		*Mod*.*1* vs *Mod*.*2*					**<0.001**

Diagnostics for regression models conducted on the proportion of sympagic OM assimilated (iPOC %) in benthic invertebrates against sea ice duration (SID) (Model 1) and SID with habitat as an additional factor (Model 2). d.f.–Degrees of freedom; Sum Sq.—Sum of squares; Mean Sq.–Mean square. Interaction between covariates is shown with a colon in Parameters. Comparison shows the result of a comparison of the model residuals using an ANOVA. P-values in bold are significant.

## 4. Discussion

Estimates of sympagic OM assimilation by the benthos were high, clearly highlighting the importance of ice-algae carbon for Arctic food-webs. Proximity to the coast influenced this assimilation of sympagic OM, as coastal samples had a generally higher contribution of sympagic carbon. Interestingly, the three target HBIs were not present in all samples, with the pelagic HBI III being particularly rare in coastal stations.

### 4.1. Distribution of HBIs

HBI presence was variable across the different compartments of the northeast Greenland shelf ecosystem. No HBIs were detected in pelagic POM samples whilst half of the sediment samples had sympagic, but no pelagic, HBIs. The faunal samples had all three target HBIs present. Previous studies that targeted the same three HBIs in pelagic POM generally detected them at low concentrations (e.g., 0.2–3.6 ng l^-1^ and 24.0±19.7 ng l^-1^ in the Canadian Arctic Archipelago [55, 56], and 0.06–3.97 ng l^-1^ in Antarctica [57]). The lack of sympagic HBIs in pelagic POM is likely due to the short residence times of sympagic organic matter in the water column [58] at the time of sampling. Sympagic HBIs are detected in the water column when sympagic production is ongoing and at the time of ice break-up and melt, when ice algae are released into the water column [55, 59]. As sympagic diatom production, the abundance of known sympagic HBI producers, and IP_25_ concentrations remained low during peak sympagic production and sedimentation in Young Sound (a coastal fjord within the study area) [59], it is probable that any detectable sympagic HBIs would already have settled out of the water column before sampling.

The distribution and phenology of the pelagic HBI III is less well-studied, but it is currently only known to be produced by members of the diatom genera *Rhizosolenia* and *Pleurosigma* [60, 61]. The water column on the northeast Greenland shelf is nitrate-poor, and the known HBI III producers constitute <1% of the phytoplankton community when present [33, 62]. Analysis of the protist community at the time of sampling showed a dominance of flagellates and dinoflagellates, with diatoms only contributing to ~5% of total community abundance (except at Station 16, where they contributed ~75%) [63]. Diatom production and dominance of the phytoplankton community in Northeast Greenland waters is usually short-lived, with very high export fluxes occurring in June to July, and very little presence in August [64–66]. Therefore, the lack of pelagic HBIs in POM samples is likely due to a very low abundance of HBI III producers throughout the study area due to the timing of sampling [64]. Additionally, as HBI III biosynthesis is likely favoured by nutrient-rich conditions [30], it may be produced in lower amounts on this nutrient-poor shelf [65].

The settling of sympagic algae and the low production of HBI III is also reflected in the low sediment H-Print values (i.e., low relative pelagic HBI content) found throughout the study area. Notably, there was a difference between habitat types, as the shelf stations (S13 and S19) had ~2–4% higher H-Print, whereas most of the coastal stations had no HBI III. This does not necessarily indicate that HBI III was absent in these locations. Terrestrial and glacial run-off can dilute OM concentrations in sediments through sedimentation of inorganic sediments and contributions of terrestrial OM. Freshwater from runoff and subglacial discharge also limit primary production in east Greenland fjords [67, 68] and can result in chrysophyte- and dinoflagellate-dominated phytoplankton communities [44, 62]. The lower H-Print in sediments of coastal stations reflects this overall lower primary production by pelagic diatoms but does not quantify the potentially higher primary production by other pelagic algal groups. In Young Sound (a fjord close to S17), IP_25_ concentrations were 11 to 60 times higher than HBI III concentrations, reflecting a similarly higher presence of sympagic HBIs over pelagic HBIs [69]. Greenland shelf waters tend to be more productive than fjords due to nutrient input from advected Atlantic Water and lower freshwater influence [70, 71]. Diatoms make up a higher proportion of the phytoplankton community there (~30% *vs*. ~80%) [62], making HBI III production more likely in shelf regions. This highlights the dependence of HBI-based methods on there being appropriate conditions (i.e. presence of HBI producers and adequate nutrient levels) for HBI production. This has implications for both ecological studies, where a robust analysis of microalgal communities needs to be carried out to determine whether HBI analyses can be used, and for paleoclimatic studies, where a lack of HBIs can simply represent inadequate growth conditions.

The lack of HBIs in many environmental samples (POM and sediment) was surprising in contrast to their presence in all faunal samples. Benthic deposit feeders and suspension feeders consume, assimilate, and therefore concentrate OM (containing HBIs) from both sediments and the water column. Faunal HBI distributions followed a similar coastal-shelf pattern as sediments. At stations where fauna were collected, H-Print was <10%, except for the two shelf stations and coastal S14 and S16 (16% and 13%, respectively). Their locations outside of fjords would allow for more mixing with shelf waters, increasing pelagic primary productivity and therefore HBI III production [44]. Additionally, S14 was shallow (82 m), likely resulting in a tighter pelagic-benthic coupling and consequently more effective transfer of pelagic OM. However, the overall low H-Prints in fauna indicated a high proportion of sympagic HBIs relative to pelagic HBIs, providing high estimates of sympagic OM assimilation.

### 4.2. Sympagic carbon assimilation by fauna

The estimated percentage of sympagic OM assimilated by fauna was high (>60%) in all samples. The average proportion of 90.0±8.4% was higher than previously found for benthic communities on other Arctic shelves [17, 18, 32], but comparable to the few data known for this shelf (from *Strongylocentrotus* spp.: 95±4.3% [72]). The East Greenland shelf has the most persistent annual ice cover of Arctic shelves given both *in situ* ice formation and export from the Arctic Ocean [43]. Since more sea ice is usually linked to proportionally more sympagic algal production [10] and hence OM assimilation [17], the high values obtained here may not be surprising. It may be argued that in this region, sympagic OM is particularly important to the benthos due to the low overall primary production [33, 73]. As sea ice restricts light availability to the water column and shortens the pelagic productive season, annual pelagic production is strongly related to the duration of the open water period [74]. Sea-ice duration in the study area is nearly year-round (~300 days), and the period of greatest pelagic productivity is therefore restricted to only a couple of months per year [74]. As coupling between pelagic primary production and pelagic secondary production can be very tight in in East Greenland fjords [74, 75], the amount of pelagic OM reaching the seafloor is likely small. In contrast, sympagic OM export to the benthos is more efficient, due to faster sinking speed and low grazer populations at the time of ice algae release [76, 77], resulting in stronger sympagic-benthic coupling than pelagic-benthic coupling.

Shelf fauna generally had a lower assimilation of sympagic OM than fjord fauna. Pelagic OM plays a larger role in the diets of shelf fauna, likely because of the higher level of pelagic primary production on the open shelf [70]. In fjords, pelagic primary production is limited by higher turbidity from run-off, higher stratification due to more freshwater input and lower wind mixing, and less nutrient input, as the water masses with high nutrient concentrations (e.g. Atlantic Water) are usually on the shelf or in deeper waters [67, 68, 71]. In addition, the large amounts of freshwater present in fjords may have restricted HBI production, further accentuating this environmental difference. HBI-producing taxa in environments with high freshwater input have been found to produce less HBIs than expected [59, 68, 78]. As HBI III production generally occurs during (or immediately following) peak melting periods [30], the freshwater input in these restricted fjord systems may lead to very low levels of HBI III production (and therefore low estimates of pelagic OM in benthic fauna). Finally, sea ice in coastal areas generally has a stronger early season (i.e. before large meltwater inputs) bloom development than offshore ice due to more stable conditions [45, 46], which is also reflected in the higher sympagic OM assimilation by coastal fauna.

HBI-based analyses only reflect OM production that occurs in the upper water column. In this system, other sources of OM are probably an important proportion of benthic diets, especially in the coastal stations. In Young Sound, benthic sources of carbon (macroalgae, microphytobenthos) contributed to ~25% of the carbon assimilated into shallow benthic food-webs, whereas terrestrial organic carbon did not contribute significantly [79]. It is unknown how much these sources contribute to benthic carbon assimilation in deeper waters, but, in the same fjord, terrestrial organic carbon constituted 40% of the POC measured in sediment traps at a depth of 65 m [65]. If these sources contribute significantly to carbon assimilated by benthos, especially in the coastal stations, the importance of sympagic carbon presented here would be lessened. For example, a nearshore study in Young Sound using fatty acids, bulk- and compound-specific isotopes, found that on average to 26% of the diet of *Tridonta borealis* came from sympagic carbon and 19% from pelagic carbon [80]. While sympagic carbon is still more important than pelagic carbon, benthic sources (microphytobenthos and macroalgae) also played an important role in that study: 22 and 33%, respectively [80]. A better understanding of these contributions throughout the environment is important to better predict how a change in ice cover will affect food delivery to the benthos, but this would require a multi-trophic marker approach.

The main factor affecting proportion of sympagic OM assimilated in this study is the stronger pelagic production on the open shelf (see previous section), especially the comparatively lower sympagic OM assimilation found at Station 2. This station is on a shallow bank, a bathymetric feature known to increase pelagic primary production and pelagic-benthic coupling [41]. The lack of a relationship between SID and proportion of sympagic OM assimilated is unexpected, as a strong relationship between these variables has been found on other Arctic shelves [17, 18]. This may be explained by the narrow range of SID values (261–311 days y^-1^), not large enough to have a discernible effect on the variability of proportion of sympagic OM assimilated (Fig 4). Previous studies, in contrast, had SID ranges spanning around 250 days y^-1^ [17, 18]. A study with a larger latitudinal or longitudinal (and therefore SID) range would permit more comparable values between inflow shelves and this outflow shelf.

Although different animal tissues were used for different taxa, this is not expected to have had an effect on the results. Other biomarkers (e.g. stable isotopes) show different turnover rates in different tissues, and this can be utilised to assess different biological mechanisms [81–83]. However, there is very little information on HBI turnover in animals, with only one study having investigated it (and finding a turnover rate of around 1 month) [17]. Since the HBIs used in this method all have very similar molecular structures, they are therefore expected to behave similarly once ingested. As HBI-based analysis relies on the ratio of HBIs, as long as they have similar turnover rates, the results should all show the same mechanism.

Estimates of sympagic OM contribution to benthic food-webs do not match what little we know about the relative contributions of sympagic and pelagic to primary production in Greenland waters. Although there are few estimates of sympagic production on the East Greenland shelf, measurements in Young Sound indicate that levels of sympagic production are <1% of the levels of annual pelagic production in the same area [73]. Sympagic production throughout the Arctic is rarely >50% of total primary production, except in the permanently covered central Arctic Ocean [10, 84]. The discrepancy between sympagic production contributing so little to total primary production, but estimated at 90% of the OM assimilated by benthos, is difficult to reconcile. This is likely due to a combination of the differential flux of sympagic and pelagic primary producers (ice algae generally sediment more rapidly, e.g., [57, 75]), grazing of pelagic OM in the water column [73, 74], possible selective feeding on sympagic OM by certain benthic organisms (e.g., [85]), and methodological limitations. The estimation method used here is based on the ratio of three HBIs and is, therefore, sensitive to variations in individual HBI concentrations. The empirical relationship between H-Print and the proportion of OM was determined in a single laboratory feeding experiment, where the sympagic HBIs and pelagic HBI represented set amounts of sympagic and pelagic carbon, respectively [31]. However, the production and degradation of HBIs is still not well understood, and these molecules are unlikely to represent such a one-to-one representation of sympagic and pelagic OM *in situ*. For example, we find that HBI III is present in low concentrations–or absent–throughout the study area at the time of sampling. As it is the only representation of pelagic OM in this estimation method (and overlooks production by non-diatom and most diatom producers), this leads to potential overestimate of sympagic carbon assimilation by consumers: while the values here potentially represent a maximum possibly contribution of sympagic OM, they are likely overestimates. Future studies should combine multiple biomarkers in the same study to allow for comparison, and to capture the full range of carbon sources available (see [86]). Additionally, studying HBI distributions in environmental samples (POM, sediment) as well as fauna, can give an indication of how reliable this method at the time and location of the study.

## 5. Conclusion

On the Northeast Greenland shelf, the signature of sympagic OM was generally high throughout the benthic ecosystem. In general, H-Print was low (<25%) and showed a dominance of sympagic HBIs. Sediment and faunal H-Print values were especially low in coastal stations, where HBI III production is likely low due to higher freshwater input, lower nutrient availability, and lower pelagic production. This has implications for the interpretation of results based on HBI analyses, as the production of HBIs is not uniform and dependent on there being adequate environmental conditions. The low H-Print found led to high estimates of sympagic OM assimilation by fauna. Even if those estimates were overly high due to methodological bias (e.g., absence of the pelagic HBI III in some samples), results indicate that sympagic production can be a major source of OM for benthic fauna on the northeast Greenland shelf compared to pelagic sources. The higher proportion of sympagic OM assimilated in coastal areas may have been due to a nutrient-restricted pelagic production after the pelagic spring bloom in these areas. Recent studies indicate that pelagic-benthic coupling in this region has weakened in the past 30 years. This was suggested to be caused by phytoplankton communities tending towards smaller cell sizes in response to nutrient poor conditions, and a stronger retention in the water column through a combination of slow-sinking small cells, strong stratification, and a more active pelagic food web [41]. Coupled with reductions in sea ice duration (and therefore sympagic OM input), this may lead to a reduction in food input to the benthos, with implications for future community structure and function. Finally, other studies from the study area suggest that terrestrial OM from glacial and snow melt run-off and benthic primary production may contribute substantial amounts of organic carbon and may represent important carbon sources beyond those studied here.

## Supporting information

S1 Fig Proportion of sympagic OM assimilated (iPOC %) by individual taxa. Estimates of sympagic OM assimilated in benthic taxa, coloured according to phylum. Horizontal dashed lines separate phyla. Boxes show the interquartile range and the vertical black line in each box is the median. Open triangles are individual data points, outliers are filled black circles. Numbers on the right are sample size. Note the restricted *x*-axis range.
(DOCX)

S1 Table Details of HBI analysis of samples of pelagic POM, sediment POM and fauna collected in East Greenland in September-August 2022. Table showing the metadata of each sample, along with the volume of filtered water for pelagic POM samples; the mass of dry sediment analysed for sediment POM samples; and the taxon, tissue analysed and mass of analysed tissue for faunal samples. The H-Print and (for faunal samples) estimated percentage of sympagic OM assimilated (iPOC %) are shown.
(DOCX)

S2 Table Post-hoc Tukey test of invertebrate H-Prints. (DOCX)

S3 Table ANOVA results for proportion of sympagic organic matter assimilated (iPOC %) by benthic invertebrates. (DOCX)

S4 Table Post-hoc Tukey test of invertebrate proportion of sympagic organic matter (iPOC %)assimilated. (DOCX)
