# Supplementary material for: High uptake of sympagic organic matter by benthos on an Arctic outflow shelf
Source: PLoS One. 2024 Aug 7;19(8):e0308562. doi: 10.1371/journal.pone.0308562 (PMC11305566; doi:10.1371/journal.pone.0308562)
Supplement: S1 Fig — Estimates of sympagic OM assimilated in benthic taxa, coloured according to phylum. Horizontal dashed lines separate phyla. Boxes show the interquartile range and the vertical black line in each box is the median. Open triangles are individual data points, outliers are filled black circles. Numbers on the right are sample size. Note the restricted x-axis range. (DOCX) [file pone.0308562.s001.docx]

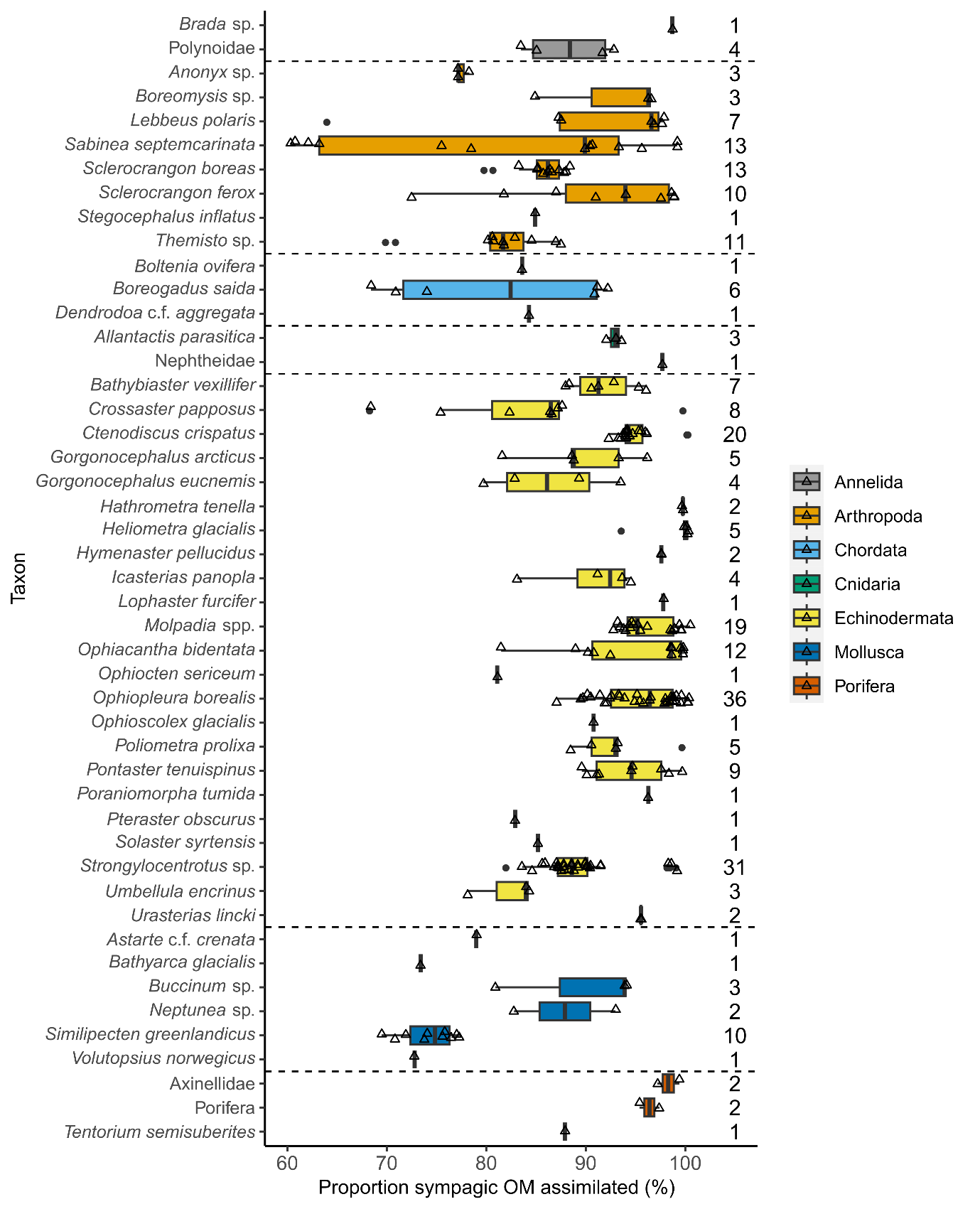


**S1 Fig.** Estimates of sympagic OM assimilated in benthic taxa, coloured according to phylum. Horizontal dashed lines separate phyla. Boxes show the interquartile range and the vertical black line in each box is the median. Open triangles are individual data points, outliers are filled black circles. Numbers on the right are sample size. Note the restricted *x*-axis range.
