# Supplementary material for: High uptake of sympagic organic matter by benthos on an Arctic outflow shelf
Source: PLoS One. 2024 Aug 7;19(8):e0308562. doi: 10.1371/journal.pone.0308562 (PMC11305566; doi:10.1371/journal.pone.0308562)
Supplement: S1 Table — Table showing the metadata of each sample, along with the volume of filtered water for pelagic POM samples; the mass of dry sediment analysed for sediment POM samples; and the taxon, tissue analysed and mass of analysed tissue for faunal samples. The H-Print and (for faunal samples) estimated percentage of sympagic OM assimilated (iPOC %) are shown. (DOCX) [file pone.0308562.s002.docx]

| **S1 Table. Details of HBI analysis of samples of pelagic POM, sediment POM and fauna collected in East Greenland in September-August 2022.** Table showing the metadata of each sample, along with the volume of filtered water for pelagic POM samples; the mass of dry sediment analysed for sediment POM samples; and the taxon, tissue analysed and mass of analysed tissue for faunal samples. The H-Print and (for faunal samples) estimated percentage of sympagic OM assimilated are shown. | | | | | | | | | | |
| --- | --- | --- | --- | --- | --- | --- | --- | --- | --- | --- |
| **Station** | **Habitat** | **Sample Type** | **Depth** | **Sea Ice Duration** | **Taxon** | **Number Of Individuals** | **Tissue Targeted** | **Mass/ Volume Analysed** | **H-Print** | **Sympagic OM %** |
| 2 | Shelf | Fauna | 70 | 308 | Polynoidae | 4 | Fragments and head | 0.6252 | 17.9 | 83.5 |
| 2 | Shelf | Fauna | 70 | 308 | Polynoidae | 2 | Fragments and head | 0.4568 | 16.3 | 85.1 |
| 2 | Shelf | Fauna | 70 | 308 | *Anonyx* sp*.* | 10 | Whole | 0.7477 | 24.1 | 77.2 |
| 2 | Shelf | Fauna | 70 | 308 | *Anonyx* sp*.* | 9 | Whole | 0.637 | 24.1 | 77.2 |
| 2 | Shelf | Fauna | 70 | 308 | *Anonyx* sp*.* | 10 | Whole | 0.7641 | 23.1 | 78.3 |
| 2 | Shelf | Fauna | 70 | 308 | *Lebbeus polaris* | 2 | Abdomen | 0.2203 | 37 | 64.1 |
| 2 | Shelf | Fauna | 70 | 308 | *Sabinea septemcarinata* | 3 | Abdomen | 0.657 | 40.7 | 60.3 |
| 2 | Shelf | Fauna | 70 | 308 | *Sabinea septemcarinata* | 3 | Abdomen | 0.9 | 40.2 | 60.8 |
| 2 | Shelf | Fauna | 70 | 308 | *Sabinea septemcarinata* | 3 | Abdomen | 0.6147 | 38.9 | 62.1 |
| 2 | Shelf | Fauna | 70 | 308 | *Sabinea septemcarinata* | 3 | Abdomen | 0.815 | 37.9 | 63.2 |
| 2 | Shelf | Fauna | 70 | 308 | *Sabinea septemcarinata* | 3 | Abdomen | 0.801 | 25.8 | 75.5 |
| 2 | Shelf | Fauna | 70 | 308 | *Sabinea septemcarinata* | 3 | Abdomen | 0.7513 | 22.9 | 78.5 |
| 2 | Shelf | Fauna | 70 | 308 | *Stegocephalus inflatus* | 4 | Whole | 0.7898 | 16.6 | 84.9 |
| 2 | Shelf | Fauna | 70 | 308 | *Themisto* sp*.* | 15 | Whole | 0.6608 | 31.2 | 70 |
| 2 | Shelf | Fauna | 70 | 308 | *Themisto* sp*.* | 15 | Whole | 0.474 | 16.9 | 84.6 |
| 2 | Shelf | Fauna | 70 | 308 | *Boreogadus saida* | 1 | Liver | 0.3331 | 32.7 | 68.4 |
| 2 | Shelf | Fauna | 70 | 308 | *Boreogadus saida* | 1 | Liver | 0.3429 | 30.3 | 70.9 |
| 2 | Shelf | Fauna | 70 | 308 | *Boreogadus saida* | 1 | Liver | 0.35 | 27.2 | 74 |
| 2 | Shelf | Fauna | 70 | 308 | *Crossaster papposus* | 1 | Whole | 0.816 | 32.8 | 68.4 |
| 2 | Shelf | Fauna | 70 | 308 | *Crossaster papposus* | 1 | Whole | 0.631 | 25.9 | 75.4 |
| 2 | Shelf | Fauna | 70 | 308 | *Crossaster papposus* | 1 | Whole | 0.714 | 19.1 | 82.3 |
| 2 | Shelf | Fauna | 70 | 308 | *Crossaster papposus* | 1 | Whole | 0.5186 | 15.1 | 86.4 |
| 2 | Shelf | Fauna | 70 | 308 | *Crossaster papposus* | 1 | Whole | 0.6178 | 14.9 | 86.6 |
| 2 | Shelf | Fauna | 70 | 308 | *Crossaster papposus* | 1 | Whole | 0.7058 | 14.3 | 87.2 |
| 2 | Shelf | Fauna | 70 | 308 | *Ophiocten sericeum* | 4 | Whole | 0.6143 | 20.3 | 81.1 |
| 2 | Shelf | Fauna | 70 | 308 | *Strongylocentrotus* sp*.* | 1 | Whole | 0.876 | 16.8 | 84.6 |
| 2 | Shelf | Fauna | 70 | 308 | *Strongylocentrotus* sp*.* | 1 | Whole | 0.7443 | 14.4 | 87.1 |
| 2 | Shelf | Fauna | 70 | 308 | *Strongylocentrotus* sp*.* | 1 | Test and stomach removed | 0.5536 | 14.3 | 87.2 |
| 2 | Shelf | Fauna | 70 | 308 | *Strongylocentrotus* sp*.* | 1 | Test and stomach removed | 0.6719 | 14.3 | 87.2 |
| 2 | Shelf | Fauna | 70 | 308 | *Strongylocentrotus* sp*.* | 1 | Test and stomach removed | 0.6821 | 13.9 | 87.6 |
| 2 | Shelf | Fauna | 70 | 308 | *Strongylocentrotus* sp*.* | 1 | Whole | 0.7516 | 13.7 | 87.8 |
| 2 | Shelf | Fauna | 70 | 308 | *Strongylocentrotus* sp*.* | 1 | Test and stomach removed | 0.7529 | 13.7 | 87.8 |
| 2 | Shelf | Fauna | 70 | 308 | *Strongylocentrotus* sp*.* | 1 | Test and stomach removed | 0.771 | 13.7 | 87.9 |
| 2 | Shelf | Fauna | 70 | 308 | *Strongylocentrotus* sp*.* | 1 | Whole | 0.7003 | 13.2 | 88.3 |
| 2 | Shelf | Fauna | 70 | 308 | *Strongylocentrotus* sp*.* | 3 | Whole | 0.7471 | 12.4 | 89.2 |
| 2 | Shelf | Fauna | 70 | 308 | *Similipecten greenlandicus* | 60 | Whole | 0.9978 | 31.7 | 69.5 |
| 2 | Shelf | Fauna | 70 | 308 | *Similipecten greenlandicus* | 60 | Whole | 0.994 | 30.4 | 70.8 |
| 2 | Shelf | Fauna | 70 | 308 | *Similipecten greenlandicus* | 60 | Shell removed | 0.69 | 29.4 | 71.9 |
| 2 | Shelf | Fauna | 70 | 308 | *Similipecten greenlandicus* | 60 | Whole | 0.993 | 27.4 | 73.8 |
| 2 | Shelf | Fauna | 70 | 308 | *Similipecten greenlandicus* | 60 | Whole | 1 | 27.2 | 74.1 |
| 2 | Shelf | Fauna | 70 | 308 | *Similipecten greenlandicus* | 60 | Whole | 0.969 | 25.7 | 75.6 |
| 2 | Shelf | Fauna | 70 | 308 | *Similipecten greenlandicus* | 60 | Whole | 0.967 | 25.5 | 75.8 |
| 2 | Shelf | Fauna | 70 | 308 | *Similipecten greenlandicus* | 60 | Whole | 0.933 | 24.8 | 76.5 |
| 2 | Shelf | Fauna | 70 | 308 | *Similipecten greenlandicus* | 60 | Whole | 0.993 | 24.3 | 77 |
| 2 | Shelf | Fauna | 70 | 308 | *Similipecten greenlandicus* | 60 | Whole | 0.979 | 24.1 | 77.3 |
| 3 | Coastal | Fauna | 226 | 294 | *Brada sp.* | 4 | Whole | 0.8013 | 3 | 98.7 |
| 3 | Coastal | Fauna | 226 | 294 | *Sabinea septemcarinata* | 1 | Abdomen | 0.6062 | 2.6 | 99.2 |
| 3 | Coastal | Fauna | 226 | 294 | *Sabinea septemcarinata* | 1 | Abdomen | 0.6082 | 2.5 | 99.2 |
| 3 | Coastal | Fauna | 226 | 294 | *Sclerocrangon ferox* | 1 | Abdomen | 0.6353 | 4.2 | 97.6 |
| 3 | Coastal | Fauna | 226 | 294 | *Sclerocrangon ferox* | 1 | Abdomen | 0.6038 | 3.1 | 98.6 |
| 3 | Coastal | Fauna | 226 | 294 | *Sclerocrangon ferox* | 1 | Whole | 0.6391 | 2.9 | 98.9 |
| 3 | Coastal | Fauna | 226 | 294 | *Sclerocrangon ferox* | 1 | Whole | 0.4668 | 2.8 | 98.9 |
| 3 | Coastal | Fauna | 226 | 294 | *Crossaster papposus* | 1 | Whole | 0.6525 | 1.8 | 99.9 |
| 3 | Coastal | Fauna | 226 | 294 | *Ctenodiscus crispatus* | 1 | Stomach removed | 0.7315 | 1.5 | 100.3 |
| 3 | Coastal | Fauna | 226 | 294 | *Ctenodiscus crispatus* | 1 | Stomach removed | 0.8106 | 1.4 | 100.3 |
| 3 | Coastal | Fauna | 226 | 294 | *Ctenodiscus crispatus* | 1 | Stomach removed | 0.6974 | 1.4 | 100.4 |
| 3 | Coastal | Fauna | 226 | 294 | *Gorgonocephalus arcticus* | 1 | Disk | 0.7195 | 5.5 | 96.2 |
| 3 | Coastal | Fauna | 226 | 294 | *Hathrometra tenella* | 6 | Disk | 0.6839 | 2 | 99.7 |
| 3 | Coastal | Fauna | 226 | 294 | *Hathrometra tenella* | 5 | Disk | 0.6286 | 2 | 99.8 |
| 3 | Coastal | Fauna | 226 | 294 | *Heliometra glacialis* | 1 | Disk | 0.4813 | 1.8 | 99.9 |
| 3 | Coastal | Fauna | 226 | 294 | *Heliometra glacialis* | 1 | Whole | 0.7335 | 1.7 | 100.1 |
| 3 | Coastal | Fauna | 226 | 294 | *Heliometra glacialis* | 1 | Disk | 0.6203 | 1.6 | 100.2 |
| 3 | Coastal | Fauna | 226 | 294 | *Heliometra glacialis* | 1 | Disk | 0.7086 | 1.5 | 100.3 |
| 3 | Coastal | Fauna | 226 | 294 | *Lophaster furcifer* | 1 | Whole | 0.7229 | 4 | 97.8 |
| 3 | Coastal | Fauna | 226 | 294 | *Molpadia* spp*.* | 1 | Stomach removed | 0.8968 | 6.2 | 95.5 |
| 3 | Coastal | Fauna | 226 | 294 | *Molpadia* spp*.* | 1 | Stomach removed | 0.5012 | 3.2 | 98.5 |
| 3 | Coastal | Fauna | 226 | 294 | *Molpadia* spp*.* | 1 | Stomach removed | 0.5056 | 2.4 | 99.4 |
| 3 | Coastal | Fauna | 226 | 294 | *Ophiacantha bidentata* | 4 | Whole | 0.4813 | 3 | 98.7 |
| 3 | Coastal | Fauna | 226 | 294 | *Ophiacantha bidentata* | 5 | Whole | 0.6487 | 2.3 | 99.5 |
| 3 | Coastal | Fauna | 226 | 294 | *Ophiacantha bidentata* | 5 | Whole | 0.6254 | 2 | 99.8 |
| 3 | Coastal | Fauna | 226 | 294 | *Ophiacantha bidentata* | 4 | Whole | 0.7021 | 2 | 99.8 |
| 3 | Coastal | Fauna | 226 | 294 | *Ophiacantha bidentata* | 5 | Whole | 0.5832 | 2 | 99.8 |
| 3 | Coastal | Fauna | 226 | 294 | *Ophiopleura borealis* | 1 | Whole | 0.6409 | 1.5 | 100.2 |
| 3 | Coastal | Fauna | 226 | 294 | *Ophiopleura borealis* | 3 | Whole | 0.7412 | 1.5 | 100.3 |
| 3 | Coastal | Fauna | 226 | 294 | *Ophiopleura borealis* | 1 | Disk | 0.6587 | 1.4 | 100.4 |
| 3 | Coastal | Fauna | 226 | 294 | *Poliometra prolixa* | 3 | Whole | 0.7094 | 2 | 99.8 |
| 3 | Coastal | Fauna | 226 | 294 | *Pontaster tenuispinus* | 1 | Whole | 0.7065 | 2 | 99.7 |
| 3 | Coastal | Fauna | 226 | 294 | Axinellidae | 1 | Fragment | 0.8254 | 2.3 | 99.4 |
| 5 | Coastal | Fauna | 447 | 311 | *Boreomysis* sp*.* | 50 | Whole | 0.4837 | 5.4 | 96.3 |
| 5 | Coastal | Fauna | 447 | 311 | *Boreomysis* sp*.* | 50 | Whole | 0.5001 | 5.1 | 96.6 |
| 5 | Coastal | Fauna | 447 | 311 | *Lebbeus polaris* | 2 | Whole | 0.5015 | 5.1 | 96.6 |
| 5 | Coastal | Fauna | 447 | 311 | *Lebbeus polaris* | 2 | Whole | 0.5913 | 4.8 | 96.9 |
| 5 | Coastal | Fauna | 447 | 311 | *Lebbeus polaris* | 2 | Whole | 0.5571 | 4 | 97.7 |
| 5 | Coastal | Fauna | 447 | 311 | *Lebbeus polaris* | 2 | Whole | 0.5848 | 3.8 | 97.9 |
| 5 | Coastal | Fauna | 447 | 311 | *Boreogadus saida* | 1 | Liver | 0.3126 | 10.7 | 90.9 |
| 5 | Coastal | Fauna | 447 | 311 | *Boreogadus saida* | 1 | Liver | 0.4137 | 10.4 | 91.2 |
| 5 | Coastal | Fauna | 447 | 311 | *Boreogadus saida* | 1 | Liver | 0.4429 | 9.4 | 92.2 |
| 5 | Coastal | Fauna | 447 | 311 | *Allantactis parasitica* | 1 | Not whole | 0.5818 | 9.5 | 92.1 |
| 5 | Coastal | Fauna | 447 | 311 | *Allantactis parasitica* | 1 | Not whole | 0.5931 | 8.1 | 93.6 |
| 5 | Coastal | Fauna | 447 | 311 | Nephtheidae | 1 | Whole | 0.5129 | 4 | 97.7 |
| 5 | Coastal | Fauna | 447 | 311 | *Gorgonocephalus arcticus* | 1 | Disk | 0.4118 | 12.7 | 88.8 |
| 5 | Coastal | Fauna | 447 | 311 | *Hymenaster pellucidus* | 1 | Whole | 0.6696 | 4.1 | 97.6 |
| 5 | Coastal | Fauna | 447 | 311 | *Hymenaster pellucidus* | 1 | Whole | 0.6863 | 4.1 | 97.6 |
| 5 | Coastal | Fauna | 447 | 311 | *Molpadia* spp*.* | 1 | Partly removed stomach | 0.6233 | 2.8 | 98.9 |
| 5 | Coastal | Fauna | 447 | 311 | *Ophiacantha bidentata* | 10 | Disk | 0.6295 | 3.2 | 98.5 |
| 5 | Coastal | Fauna | 447 | 311 | *Ophiacantha bidentata* | 10 | Disk | 0.6546 | 3.1 | 98.6 |
| 5 | Coastal | Fauna | 447 | 311 | *Ophiopleura borealis* | 1 | Whole | 0.6679 | 14.4 | 87.1 |
| 5 | Coastal | Fauna | 447 | 311 | *Ophiopleura borealis* | 1 | Whole | 0.8169 | 8.4 | 93.3 |
| 5 | Coastal | Fauna | 447 | 311 | *Ophiopleura borealis* | 1 | Whole | 0.7512 | 6.5 | 95.2 |
| 5 | Coastal | Fauna | 447 | 311 | *Ophiopleura borealis* | 1 | Whole | 0.8009 | 3 | 98.7 |
| 5 | Coastal | Fauna | 447 | 311 | *Ophiopleura borealis* | 1 | Disk | 0.6995 | 3 | 98.8 |
| 5 | Coastal | Fauna | 447 | 311 | *Ophiopleura borealis* | 1 | Whole | 0.7724 | 3 | 98.8 |
| 5 | Coastal | Fauna | 447 | 311 | *Ophiopleura borealis* | 1 | Whole | 0.7292 | 2.2 | 99.6 |
| 5 | Coastal | Fauna | 447 | 311 | *Pontaster tenuispinus* | 4 | Whole | 0.8555 | 3.3 | 98.4 |
| 5 | Coastal | Fauna | 447 | 311 | *Poraniomorpha tumida* | 1 | Whole | 0.6229 | 5.3 | 96.3 |
| 5 | Coastal | Fauna | 447 | 311 | *Urasterias lincki* | 1 | Whole | 0.8055 | 6.1 | 95.6 |
| 5 | Coastal | Fauna | 447 | 311 | *Neptunea* sp*.* | 1 | Foot | 0.7369 | 8.6 | 93 |
| 5 | Coastal | Fauna | 447 | 311 | Axinellidae | 1 | Fragment | 1.0593 | 4.6 | 97.2 |
| 5 | Coastal | Fauna | 447 | 311 | Porifera | 1 | Whole | 0.7778 | 6.3 | 95.4 |
| 9 | Coastal | Fauna | 231 | 311 | *Gorgonocephalus arcticus* | 1 | Disk | 0.3372 | 12.9 | 88.6 |
| 9 | Coastal | Fauna | 231 | 311 | *Gorgonocephalus arcticus* | 1 | Disk | 0.4772 | 8.3 | 93.3 |
| 9 | Coastal | Fauna | 231 | 311 | *Icasterias panopla* | 1 | Part | 0.572 | 8 | 93.7 |
| 9 | Coastal | Fauna | 231 | 311 | *Icasterias panopla* | 1 | Part | 0.6084 | 7.1 | 94.5 |
| 9 | Coastal | Fauna | 231 | 311 | *Molpadia* spp*.* | 1 | Stomach removed | 0.6066 | 2.2 | 99.6 |
| 9 | Coastal | Fauna | 231 | 311 | *Molpadia* spp*.* | 1 | Stomach removed | 0.5607 | 2.2 | 99.6 |
| 9 | Coastal | Fauna | 231 | 311 | *Ophiopleura borealis* | 1 | Disk | 0.619 | 11.1 | 90.5 |
| 9 | Coastal | Fauna | 231 | 311 | *Ophiopleura borealis* | 1 | Disk | 0.6262 | 3.8 | 97.9 |
| 9 | Coastal | Fauna | 231 | 311 | *Ophiopleura borealis* | 1 | Disk | 0.6207 | 3.6 | 98.2 |
| 9 | Coastal | Fauna | 231 | 311 | *Ophiopleura borealis* | 1 | Disk | 0.7404 | 3.5 | 98.2 |
| 9 | Coastal | Fauna | 231 | 311 | *Ophiopleura borealis* | 1 | Disk | 0.7076 | 3.3 | 98.5 |
| 9 | Coastal | Fauna | 231 | 311 | *Ophiopleura borealis* | 1 | Disk | 0.7572 | 3.1 | 98.6 |
| 9 | Coastal | Fauna | 231 | 311 | *Ophiopleura borealis* | 1 | Disk | 0.6336 | 3 | 98.7 |
| 9 | Coastal | Fauna | 231 | 311 | *Ophiopleura borealis* | 1 | Disk | 0.7115 | 2.6 | 99.1 |
| 9 | Coastal | Fauna | 231 | 311 | *Ophiopleura borealis* | 1 | Disk | 0.7405 | 2.6 | 99.1 |
| 9 | Coastal | Fauna | 231 | 311 | *Ophiopleura borealis* | 1 | Disk | 0.5464 | 2.1 | 99.6 |
| 9 | Coastal | Fauna | 231 | 311 | *Pontaster tenuispinus* | 3 | Whole | 0.679 | 4.1 | 97.6 |
| 9 | Coastal | Fauna | 231 | 311 | *Strongylocentrotus* sp*.* | 1 | Test and stomach removed | 0.7271 | 3.4 | 98.3 |
| 9 | Coastal | Fauna | 231 | 311 | *Strongylocentrotus* sp*.* | 1 | Whole | 0.6864 | 3.2 | 98.6 |
| 9 | Coastal | Fauna | 231 | 311 | *Strongylocentrotus* sp*.* | 1 | Test and stomach removed | 0.9007 | 2.6 | 99.2 |
| 9 | Coastal | Fauna | 231 | 311 | *Urasterias lincki* | 1 | Part of disk, 1 arm | 0.5224 | 6.1 | 95.5 |
| 9 | Coastal | Fauna | 231 | 311 | *Buccinum* sp*.* | 1 | Mantle and foot separate | 0.6872 | 7.8 | 93.9 |
| 9 | Coastal | Fauna | 231 | 311 | *Buccinum* sp. | 1 | Mantle and foot separate | 0.8301 | 7.6 | 94.1 |
| 9 | Coastal | Fauna | 231 | 311 | *Porifera* | 1 | Part | 0.7491 | 4.3 | 97.4 |
| 14 | Coastal | Fauna | 82 | 289 | *Polynoidae* | 1 | Whole | 0.5234 | 9.9 | 91.7 |
| 14 | Coastal | Fauna | 82 | 289 | *Polynoidae* | 1 | Whole | 0.7592 | 8.9 | 92.8 |
| 14 | Coastal | Fauna | 82 | 289 | *Sclerocrangon boreas* | 1 | Abdomen | 0.2976 | 20.6 | 80.8 |
| 14 | Coastal | Fauna | 82 | 289 | *Sclerocrangon boreas* | 1 | Abdomen | 0.3925 | 15.7 | 85.7 |
| 14 | Coastal | Fauna | 82 | 289 | *Boltenia ovifera* | 1 | Stalk removed | 0.7108 | 17.9 | 83.6 |
| 14 | Coastal | Fauna | 82 | 289 | *Dendrodoa* c*.*f*. aggregata* | 20 | Whole | 0.7867 | 17.2 | 84.3 |
| 14 | Coastal | Fauna | 82 | 289 | *Gorgonocephalus eucnemis* | 1 | Disk | 0.3913 | 21.7 | 79.7 |
| 14 | Coastal | Fauna | 82 | 289 | *Gorgonocephalus eucnemis* | 1 | Disk | 0.434 | 18.5 | 82.9 |
| 14 | Coastal | Fauna | 82 | 289 | *Ophiopleura borealis* | 2 | Disk | 0.7335 | 9.5 | 92.1 |
| 14 | Coastal | Fauna | 82 | 289 | *Ophiopleura borealis* | 2 | Disk | 0.7243 | 9.2 | 92.4 |
| 14 | Coastal | Fauna | 82 | 289 | *Ophiopleura borealis* | 2 | Disk | 0.6849 | 9 | 92.6 |
| 14 | Coastal | Fauna | 82 | 289 | *Ophiopleura borealis* | 2 | Disk | 0.548 | 7.7 | 93.9 |
| 14 | Coastal | Fauna | 82 | 289 | *Pteraster obscurus* | 1 | Whole | 0.6436 | 18.5 | 82.9 |
| 14 | Coastal | Fauna | 82 | 289 | *Solaster syrtensis* | 1 | Whole | 0.7368 | 16.3 | 85.2 |
| 14 | Coastal | Fauna | 82 | 289 | *Strongylocentrotus* sp*.* | 5 | Whole | 0.896 | 10.1 | 91.5 |
| 14 | Coastal | Fauna | 82 | 289 | *Astarte* c.f. *crenata* | 40 | Whole | 0.4033 | 22.4 | 79 |
| 14 | Coastal | Fauna | 82 | 289 | *Bathyarca glacialis* | 10 | Shell removed | 0.4891 | 27.8 | 73.4 |
| 14 | Coastal | Fauna | 82 | 289 | *Buccinum* sp*.* | 1 | Mantle and foot separate | 0.7232 | 20.5 | 80.9 |
| 14 | Coastal | Fauna | 82 | 289 | *Tentorium semisuberites* | 5 | Whole | 0.8658 | 13.6 | 87.9 |
| 15 | Coastal | Fauna | 563 | 287 | *Sclerocrangon ferox* | 1 | Abdomen | 0.4748 | 10.6 | 91 |
| 15 | Coastal | Fauna | 563 | 287 | *Sclerocrangon ferox* | 1 | Abdomen | 0.6263 | 7.7 | 94 |
| 15 | Coastal | Fauna | 563 | 287 | *Sclerocrangon ferox* | 1 | Abdomen | 0.6984 | 7.6 | 94 |
| 15 | Coastal | Fauna | 563 | 287 | *Allantactis parasitica* | 1 | Whole | 0.7269 | 8.7 | 93 |
| 15 | Coastal | Fauna | 563 | 287 | *Bathybiaster vexillifer* | 1 | Whole | 0.7815 | 6.4 | 95.3 |
| 15 | Coastal | Fauna | 563 | 287 | *Bathybiaster vexillifer* | 1 | Whole | 0.701 | 5.6 | 96.1 |
| 15 | Coastal | Fauna | 563 | 287 | *Gorgonocephalus eucnemis* | 1 | Disk | 0.398 | 12.3 | 89.3 |
| 15 | Coastal | Fauna | 563 | 287 | *Gorgonocephalus eucnemis* | 2 | Disk | 0.4197 | 8.2 | 93.5 |
| 15 | Coastal | Fauna | 563 | 287 | *Ophiopleura borealis* | 2 | Disk | 0.563 | 5.9 | 95.8 |
| 15 | Coastal | Fauna | 563 | 287 | *Ophiopleura borealis* | 2 | Disk | 0.4383 | 5.3 | 96.4 |
| 15 | Coastal | Fauna | 563 | 287 | *Ophioscolex glacialis* | 3 | Whole | 0.7182 | 10.8 | 90.8 |
| 16 | Coastal | Fauna | 343 | 295 | *Boreomysis* sp*.* | 50 | Whole | 0.344 | 16.6 | 84.9 |
| 16 | Coastal | Fauna | 343 | 295 | *Sclerocrangon ferox* | 1 | Abdomen | 0.5869 | 28.7 | 72.5 |
| 16 | Coastal | Fauna | 343 | 295 | *Sclerocrangon ferox* | 1 | Abdomen | 0.6142 | 19.6 | 81.8 |
| 16 | Coastal | Fauna | 343 | 295 | *Sclerocrangon ferox* | 1 | Abdomen | 0.6415 | 14.5 | 87 |
| 16 | Coastal | Fauna | 343 | 295 | *Themisto* sp*.* | 21 | Whole | 0.3378 | 20.7 | 80.6 |
| 16 | Coastal | Fauna | 343 | 295 | *Bathybiaster vexillifer* | 1 | Stomach removed | 0.6729 | 13.6 | 88 |
| 16 | Coastal | Fauna | 343 | 295 | *Bathybiaster vexillifer* | 1 | Stomach removed | 0.7489 | 11 | 90.6 |
| 16 | Coastal | Fauna | 343 | 295 | *Ctenodiscus crispatus* | 1 | Stomach removed | 0.647 | 9.3 | 92.3 |
| 16 | Coastal | Fauna | 343 | 295 | *Ctenodiscus crispatus* | 1 | Stomach removed | 0.6482 | 8.3 | 93.3 |
| 16 | Coastal | Fauna | 343 | 295 | *Ctenodiscus crispatus* | 1 | Stomach removed | 0.7992 | 8.1 | 93.5 |
| 16 | Coastal | Fauna | 343 | 295 | *Ctenodiscus crispatus* | 1 | Stomach removed | 0.7356 | 7.7 | 94 |
| 16 | Coastal | Fauna | 343 | 295 | *Ctenodiscus crispatus* | 1 | Stomach removed | 0.6893 | 7.6 | 94.1 |
| 16 | Coastal | Fauna | 343 | 295 | *Ctenodiscus crispatus* | 1 | Stomach removed | 0.6769 | 7.5 | 94.2 |
| 16 | Coastal | Fauna | 343 | 295 | *Ctenodiscus crispatus* | 1 | Stomach removed | 0.7591 | 7.4 | 94.2 |
| 16 | Coastal | Fauna | 343 | 295 | *Ctenodiscus crispatus* | 1 | Stomach removed | 0.7174 | 5.7 | 96 |
| 16 | Coastal | Fauna | 343 | 295 | *Gorgonocephalus arcticus* | 1 | Disk | 0.3918 | 19.8 | 81.6 |
| 16 | Coastal | Fauna | 343 | 295 | *Icasterias panopla* | 1 | Whole | 0.6889 | 18.3 | 83.1 |
| 16 | Coastal | Fauna | 343 | 295 | *Icasterias panopla* | 1 | Whole | 0.584 | 10.4 | 91.2 |
| 16 | Coastal | Fauna | 343 | 295 | *Molpadia* spp*.* | 1 | Stomach removed | 0.6174 | 3 | 98.7 |
| 16 | Coastal | Fauna | 343 | 295 | *Ophiacantha bidentata* | 6 | Whole | 0.5901 | 11.4 | 90.2 |
| 16 | Coastal | Fauna | 343 | 295 | *Ophiacantha bidentata* | 6 | Whole | 0.7175 | 9.1 | 92.5 |
| 16 | Coastal | Fauna | 343 | 295 | *Ophiopleura borealis* | 2 | Disk | 0.6055 | 11.9 | 89.7 |
| 16 | Coastal | Fauna | 343 | 295 | *Ophiopleura borealis* | 2 | Disk | 0.6644 | 10.2 | 91.4 |
| 16 | Coastal | Fauna | 343 | 295 | *Ophiopleura borealis* | 2 | Disk | 0.7283 | 9.7 | 91.9 |
| 16 | Coastal | Fauna | 343 | 295 | *Pontaster tenuispinus* | 2 | Whole | 0.7319 | 12 | 89.6 |
| 16 | Coastal | Fauna | 343 | 295 | *Pontaster tenuispinus* | 1 | Whole | 0.7856 | 10.3 | 91.3 |
| 16 | Coastal | Fauna | 343 | 295 | *Umbellula encrinus* | 1 | No stalk | 0.7712 | 23.2 | 78.1 |
| 16 | Coastal | Fauna | 343 | 295 | *Umbellula encrinus* | 1 | No stalk | 0.5965 | 17.4 | 84 |
| 16 | Coastal | Fauna | 343 | 295 | *Umbellula encrinus* | 1 | No stalk | 0.7536 | 17.2 | 84.3 |
| 16 | Coastal | Fauna | 343 | 295 | *Neptunea* sp. | 1 | Mantle and foot separate | 0.7154 | 18.7 | 82.8 |
| 17 | Coastal | Fauna | 216 | 279 | *Lebbeus polaris* | 5 | Abdomen | 0.7236 | 14 | 87.5 |
| 17 | Coastal | Fauna | 216 | 279 | *Bathybiaster vexillifer* | 1 | Whole | 0.892 | 13.2 | 88.3 |
| 17 | Coastal | Fauna | 216 | 279 | *Bathybiaster vexillifer* | 1 | Whole | 0.7768 | 10.3 | 91.3 |
| 17 | Coastal | Fauna | 216 | 279 | *Bathybiaster vexillifer* | 1 | Whole | 0.7093 | 8.8 | 92.8 |
| 17 | Coastal | Fauna | 216 | 279 | *Ctenodiscus crispatus* | 1 | Stomach removed | 0.691 | 7.9 | 93.8 |
| 17 | Coastal | Fauna | 216 | 279 | *Ctenodiscus crispatus* | 1 | Stomach removed | 0.6655 | 7.6 | 94 |
| 17 | Coastal | Fauna | 216 | 279 | *Ctenodiscus crispatus* | 1 | Stomach removed | 0.8273 | 7.6 | 94.1 |
| 17 | Coastal | Fauna | 216 | 279 | *Ctenodiscus crispatus* | 1 | Stomach removed | 0.7345 | 7.3 | 94.4 |
| 17 | Coastal | Fauna | 216 | 279 | *Ctenodiscus crispatus* | 1 | Stomach removed | 0.7117 | 7.2 | 94.4 |
| 17 | Coastal | Fauna | 216 | 279 | *Ctenodiscus crispatus* | 1 | Stomach removed | 0.7452 | 7 | 94.7 |
| 17 | Coastal | Fauna | 216 | 279 | *Ctenodiscus crispatus* | 1 | Stomach removed | 0.6928 | 6.3 | 95.3 |
| 17 | Coastal | Fauna | 216 | 279 | *Ctenodiscus crispatus* | 1 | Stomach removed | 0.7268 | 6.1 | 95.6 |
| 17 | Coastal | Fauna | 216 | 279 | *Ctenodiscus crispatus* | 1 | Stomach removed | 0.6024 | 5.5 | 96.1 |
| 17 | Coastal | Fauna | 216 | 279 | *Heliometra glacialis* | 1 | Peduncle and 2 arms | 0.8274 | 7.9 | 93.7 |
| 17 | Coastal | Fauna | 216 | 279 | *Molpadia* spp*.* | 1 | Stomach removed | 0.3681 | 8.8 | 92.8 |
| 17 | Coastal | Fauna | 216 | 279 | *Molpadia* spp*.* | 1 | Stomach removed | 0.3594 | 8.4 | 93.2 |
| 17 | Coastal | Fauna | 216 | 279 | *Molpadia* spp*.* | 1 | Stomach removed | 0.4518 | 8.3 | 93.4 |
| 17 | Coastal | Fauna | 216 | 279 | *Molpadia* spp*.* | 1 | Stomach removed | 0.5479 | 7.8 | 93.8 |
| 17 | Coastal | Fauna | 216 | 279 | *Molpadia* spp*.* | 1 | Stomach removed | 0.3846 | 7.7 | 94 |
| 17 | Coastal | Fauna | 216 | 279 | *Molpadia* spp*.* | 1 | Stomach removed | 0.4502 | 7.2 | 94.4 |
| 17 | Coastal | Fauna | 216 | 279 | *Molpadia* spp*.* | 1 | Stomach removed | 0.4042 | 7.1 | 94.5 |
| 17 | Coastal | Fauna | 216 | 279 | *Molpadia* spp*.* | 1 | Stomach removed | 0.4436 | 7 | 94.6 |
| 17 | Coastal | Fauna | 216 | 279 | *Molpadia* spp*.* | 1 | Stomach removed | 0.3642 | 6.5 | 95.2 |
| 17 | Coastal | Fauna | 216 | 279 | *Molpadia* spp*.* | 1 | Stomach removed | 0.4367 | 1.3 | 100.5 |
| 17 | Coastal | Fauna | 216 | 279 | *Ophiopleura borealis* | 2 | Disk | 0.7916 | 12.1 | 89.5 |
| 17 | Coastal | Fauna | 216 | 279 | *Ophiopleura borealis* | 2 | Disk | 0.7409 | 11.4 | 90.2 |
| 17 | Coastal | Fauna | 216 | 279 | *Ophiopleura borealis* | 2 | Disk | 0.7307 | 8.3 | 93.4 |
| 17 | Coastal | Fauna | 216 | 279 | *Ophiopleura borealis* | 2 | Disk | 0.665 | 6.8 | 94.9 |
| 17 | Coastal | Fauna | 216 | 279 | *Poliometra prolixa* | 10 | Peduncle | 0.8574 | 11 | 90.6 |
| 17 | Coastal | Fauna | 216 | 279 | *Poliometra prolixa* | 10 | Peduncle | 0.8607 | 8.6 | 93 |
| 17 | Coastal | Fauna | 216 | 279 | *Poliometra prolixa* | 10 | Peduncle | 0.8992 | 8.5 | 93.2 |
| 17 | Coastal | Fauna | 216 | 279 | *Pontaster tenuispinus* | 1 | Whole | 0.7527 | 11.5 | 90.1 |
| 18 | Shelf | Fauna | 210 | 261 | *Lebbeus polaris* | 15 | Abdomen | 0.645 | 14.3 | 87.3 |
| 18 | Shelf | Fauna | 210 | 261 | *Sabinea septemcarinata* | 2 | Abdomen | 0.704 | 11.7 | 89.9 |
| 18 | Shelf | Fauna | 210 | 261 | *Sabinea septemcarinata* | 2 | Abdomen | 0.6208 | 11.2 | 90.4 |
| 18 | Shelf | Fauna | 210 | 261 | *Sabinea septemcarinata* | 2 | Abdomen | 0.6592 | 10.8 | 90.7 |
| 18 | Shelf | Fauna | 210 | 261 | *Sabinea septemcarinata* | 2 | Abdomen | 0.755 | 8.4 | 93.3 |
| 18 | Shelf | Fauna | 210 | 261 | *Sabinea septemcarinata* | 2 | Abdomen | 0.764 | 6.1 | 95.6 |
| 18 | Shelf | Fauna | 210 | 261 | *Sclerocrangon boreas* | 1 | Abdomen | 0.6115 | 21.5 | 79.9 |
| 18 | Shelf | Fauna | 210 | 261 | *Sclerocrangon boreas* | 1 | Abdomen | 0.7171 | 18.1 | 83.3 |
| 18 | Shelf | Fauna | 210 | 261 | *Sclerocrangon boreas* | 1 | Abdomen | 0.8537 | 16.4 | 85.1 |
| 18 | Shelf | Fauna | 210 | 261 | *Sclerocrangon boreas* | 1 | Abdomen | 0.8114 | 16.2 | 85.2 |
| 18 | Shelf | Fauna | 210 | 261 | *Sclerocrangon boreas* | 1 | Abdomen | 0.8171 | 15.2 | 86.2 |
| 18 | Shelf | Fauna | 210 | 261 | *Sclerocrangon boreas* | 1 | Abdomen | 0.6864 | 15.1 | 86.4 |
| 18 | Shelf | Fauna | 210 | 261 | *Sclerocrangon boreas* | 1 | Abdomen | 0.6888 | 14.9 | 86.6 |
| 18 | Shelf | Fauna | 210 | 261 | *Sclerocrangon boreas* | 1 | Abdomen | 0.7892 | 14.2 | 87.3 |
| 18 | Shelf | Fauna | 210 | 261 | *Sclerocrangon boreas* | 1 | Abdomen | 0.7291 | 13.7 | 87.8 |
| 18 | Shelf | Fauna | 210 | 261 | *Sclerocrangon boreas* | 1 | Abdomen | 0.7775 | 13.5 | 88 |
| 18 | Shelf | Fauna | 210 | 261 | *Sclerocrangon boreas* | 1 | Abdomen | 0.7968 | 13.1 | 88.4 |
| 18 | Shelf | Fauna | 210 | 261 | *Themisto* sp*.* | 30 | Whole | 0.855 | 30.2 | 71 |
| 18 | Shelf | Fauna | 210 | 261 | *Themisto* sp*.* | 30 | Whole | 0.4286 | 21.2 | 80.2 |
| 18 | Shelf | Fauna | 210 | 261 | *Themisto* sp*.* | 30 | Whole | 0.352 | 20.7 | 80.7 |
| 18 | Shelf | Fauna | 210 | 261 | *Themisto* sp*.* | 30 | Whole | 0.4074 | 19.7 | 81.7 |
| 18 | Shelf | Fauna | 210 | 261 | *Themisto* sp*.* | 30 | Whole | 0.4005 | 19.6 | 81.8 |
| 18 | Shelf | Fauna | 210 | 261 | *Themisto* sp*.* | 30 | Whole | 0.3919 | 18.6 | 82.9 |
| 18 | Shelf | Fauna | 210 | 261 | *Themisto* sp*.* | 30 | Whole | 0.4046 | 14.5 | 87 |
| 18 | Shelf | Fauna | 210 | 261 | *Themisto* sp*.* | 30 | Whole | 0.3665 | 14 | 87.5 |
| 18 | Shelf | Fauna | 210 | 261 | *Crossaster papposus* | 1 | Whole | 0.6796 | 13.9 | 87.6 |
| 18 | Shelf | Fauna | 210 | 261 | *Molpadia* spp*.* | 1 | Stomach removed | 0.752 | 6.8 | 94.8 |
| 18 | Shelf | Fauna | 210 | 261 | *Molpadia* spp*.* | 1 | Stomach removed | 0.991 | 5.5 | 96.2 |
| 18 | Shelf | Fauna | 210 | 261 | *Ophiacantha bidentata* | 12 | Whole | 0.602 | 19.9 | 81.5 |
| 18 | Shelf | Fauna | 210 | 261 | *Ophiacantha bidentata* | 10 | Whole | 0.6756 | 12.5 | 89 |
| 18 | Shelf | Fauna | 210 | 261 | *Ophiacantha bidentata* | 10 | Whole | 0.652 | 10.8 | 90.8 |
| 18 | Shelf | Fauna | 210 | 261 | *Ophiopleura borealis* | 2 | Disk | 0.818 | 6.3 | 95.4 |
| 18 | Shelf | Fauna | 210 | 261 | *Ophiopleura borealis* | 2 | Disk | 0.7832 | 5.2 | 96.5 |
| 18 | Shelf | Fauna | 210 | 261 | *Ophiopleura borealis* | 2 | Disk | 0.724 | 3.8 | 98 |
| 18 | Shelf | Fauna | 210 | 261 | *Poliometra prolixa* | 8 | Whole | 0.7063 | 13 | 88.5 |
| 18 | Shelf | Fauna | 210 | 261 | *Pontaster tenuispinus* | 1 | Whole | 0.704 | 10.4 | 91.1 |
| 18 | Shelf | Fauna | 210 | 261 | *Pontaster tenuispinus* | 1 | Whole | 0.6297 | 7.1 | 94.6 |
| 18 | Shelf | Fauna | 210 | 261 | *Pontaster tenuispinus* | 1 | Whole | 0.704 | 7 | 94.7 |
| 18 | Shelf | Fauna | 210 | 261 | *Strongylocentrotus* sp*.* | 1 | Whole | 0.69 | 19.3 | 82.1 |
| 18 | Shelf | Fauna | 210 | 261 | *Strongylocentrotus* sp*.* | 1 | Whole | 0.711 | 17.9 | 83.6 |
| 18 | Shelf | Fauna | 210 | 261 | *Strongylocentrotus* sp*.* | 1 | Whole | 0.8128 | 15.9 | 85.6 |
| 18 | Shelf | Fauna | 210 | 261 | *Strongylocentrotus* sp*.* | 1 | Whole | 0.9623 | 15.5 | 85.9 |
| 18 | Shelf | Fauna | 210 | 261 | *Strongylocentrotus* sp*.* | 1 | Whole | 0.8124 | 14.7 | 86.8 |
| 18 | Shelf | Fauna | 210 | 261 | *Strongylocentrotus* sp*.* | 1 | Whole | 0.8812 | 14.2 | 87.3 |
| 18 | Shelf | Fauna | 210 | 261 | *Strongylocentrotus* sp*.* | 1 | Whole | 0.8235 | 12.9 | 88.6 |
| 18 | Shelf | Fauna | 210 | 261 | *Strongylocentrotus* sp*.* | 1 | Whole | 0.8337 | 12.9 | 88.6 |
| 18 | Shelf | Fauna | 210 | 261 | *Strongylocentrotus* sp*.* | 1 | Whole | 0.9317 | 12.8 | 88.8 |
| 18 | Shelf | Fauna | 210 | 261 | *Strongylocentrotus* sp*.* | 1 | Whole | 0.849 | 12.4 | 89.2 |
| 18 | Shelf | Fauna | 210 | 261 | *Strongylocentrotus* sp. | 1 | Whole | 0.8037 | 11.7 | 89.9 |
| 18 | Shelf | Fauna | 210 | 261 | *Strongylocentrotus* sp*.* | 1 | Whole | 0.8895 | 11.6 | 90 |
| 18 | Shelf | Fauna | 210 | 261 | *Strongylocentrotus* sp*.* | 1 | Whole | 0.9081 | 11.4 | 90.1 |
| 18 | Shelf | Fauna | 210 | 261 | *Strongylocentrotus* sp*.* | 1 | Whole | 0.7874 | 11.3 | 90.2 |
| 18 | Shelf | Fauna | 210 | 261 | *Strongylocentrotus* sp*.* | 1 | Whole | 0.922 | 11.2 | 90.4 |
| 18 | Shelf | Fauna | 210 | 261 | *Strongylocentrotus* sp*.* | 2 | Whole | 0.8207 | 11.1 | 90.5 |
| 18 | Shelf | Fauna | 210 | 261 | *Strongylocentrotus* sp*.* | 2 | Whole | 0.8187 | 10.1 | 91.5 |
| 18 | Shelf | Fauna | 210 | 261 | *Volutopsius norwegicus* | 1 | Foot | 0.7809 | 28.4 | 72.8 |
| 2 | Shelf | Pelagic POM | 32 | 308 | N/A | N/A | N/A | 5500 | N/A | N/A |
| 3 | Coastal | Pelagic POM | 21 | 294 | N/A | N/A | N/A | 6500 | N/A | N/A |
| 5 | Coastal | Pelagic POM | 34 | 311 | N/A | N/A | N/A | 7800 | N/A | N/A |
| 13 | Shelf | Pelagic POM | 43 | 274 | N/A | N/A | N/A | 12000 | N/A | N/A |
| 15 | Coastal | Pelagic POM | 38 | 287 | N/A | N/A | N/A | 8900 | N/A | N/A |
| 16 | Coastal | Pelagic POM | 28 | 295 | N/A | N/A | N/A | 12000 | N/A | N/A |
| 17 | Coastal | Pelagic POM | 30 | 279 | N/A | N/A | N/A | 12000 | N/A | N/A |
| 18 | Shelf | Pelagic POM | 45 | 261 | N/A | N/A | N/A | 11900 | N/A | N/A |
| 3 | Coastal | Sediment | 226 | 294 | N/A | N/A | N/A | 3.129 | N/A | N/A |
| 4 | Coastal | Sediment | 411 | 300 | N/A | N/A | N/A | 3.0826 | 1.9 | N/A |
| 6 | Coastal | Sediment | 444 | 311 | N/A | N/A | N/A | 3.0762 | N/A | N/A |
| 7 | Coastal | Sediment | 371 | 311 | N/A | N/A | N/A | 2.8819 | N/A | N/A |
| 9 | Coastal | Sediment | 231 | 311 | N/A | N/A | N/A | 3.1064 | 3.3 | N/A |
| 13 | Shelf | Sediment | 342 | 274 | N/A | N/A | N/A | 2.9632 | 6.4 | N/A |
| 15 | Coastal | Sediment | 563 | 287 | N/A | N/A | N/A | 2.9806 | N/A | N/A |
| 16 | Coastal | Sediment | 343 | 295 | N/A | N/A | N/A | 2.987 | 3.9 | N/A |
| 17 | Coastal | Sediment | 416 | 279 | N/A | N/A | N/A | 3.1248 | N/A | N/A |
| 19 | Shelf | Sediment | 467 | 263 | N/A | N/A | N/A | 2.9233 | 6.7 | N/A |
