# Supplementary material for: High uptake of sympagic organic matter by benthos on an Arctic outflow shelf
Source: PLoS One. 2024 Aug 7;19(8):e0308562. doi: 10.1371/journal.pone.0308562 (PMC11305566; doi:10.1371/journal.pone.0308562)
Supplement: S2 Table — (DOCX) [file pone.0308562.s003.docx]

**S2 Table**. Results of a post-hoc Tukey HSD comparing the mean H-Prints of benthic invertebrates between stations from northeast Greenland. Columns *i* and *j* show the stations being compared, while Difference shows the difference in means between those stations. Lower and upper CI are the 95% confidence intervals for the estimated difference in means.

| **Station mean compared** | | **Difference** |  |  |  |
| --- | --- | --- | --- | --- | --- |
| ***i*** | ***j*** | ***i-j*** | **Lower CI** | **Upper CI** | **p-value** |
| 3 | 2 | -21.034 | -24.645 | -17.422 | **<0.001** |
| 5 | 2 | -17.523 | -21.202 | -13.845 | **<0.001** |
| 9 | 2 | -18.554 | -22.537 | -14.570 | **<0.001** |
| 14 | 2 | -7.965 | -12.277 | -3.653 | **<0.001** |
| 15 | 2 | -15.391 | -20.692 | -10.090 | **<0.001** |
| 16 | 2 | -10.528 | -14.242 | -6.813 | **<0.001** |
| 17 | 2 | -15.210 | -18.854 | -11.565 | **<0.001** |
| 18 | 2 | -9.734 | -12.889 | -6.579 | **<0.001** |
| 5 | 3 | 3.510 | -0.432 | 7.452 | 0.125 |
| 9 | 3 | 2.480 | -1.748 | 6.708 | 0.659 |
| 14 | 3 | 13.069 | 8.530 | 17.607 | **<0.001** |
| 15 | 3 | 5.642 | 0.155 | 11.129 | **0.039** |
| 16 | 3 | 10.506 | 6.530 | 14.481 | **<0.001** |
| 17 | 3 | 5.824 | 1.914 | 9.734 | **<0.001** |
| 18 | 3 | 11.300 | 7.841 | 14.758 | **<0.001** |
| 9 | 5 | -1.030 | -5.315 | 3.255 | 0.998 |
| 14 | 5 | 9.559 | 4.967 | 14.150 | **<0.001** |
| 15 | 5 | 2.132 | -3.399 | 7.663 | 0.955 |
| 16 | 5 | 6.996 | 2.959 | 11.032 | **<0.001** |
| 17 | 5 | 2.314 | -1.658 | 6.285 | 0.668 |
| 18 | 5 | 7.789 | 4.261 | 11.318 | **<0.001** |
| 14 | 9 | 10.589 | 5.749 | 15.428 | **<0.001** |
| 15 | 9 | 3.162 | -2.576 | 8.901 | 0.732 |
| 16 | 9 | 8.026 | 3.710 | 12.342 | **<0.001** |
| 17 | 9 | 3.344 | -0.912 | 7.599 | 0.258 |
| 18 | 9 | 8.820 | 4.975 | 12.665 | **<0.001** |
| 15 | 14 | -7.426 | -13.397 | -1.455 | **0.004** |
| 16 | 14 | -2.563 | -7.184 | 2.058 | 0.725 |
| 17 | 14 | -7.245 | -11.810 | -2.681 | **<0.001** |
| 18 | 14 | -1.769 | -5.953 | 2.415 | 0.924 |
| 16 | 15 | 4.863 | -0.692 | 10.418 | 0.140 |
| 17 | 15 | 0.181 | -5.327 | 5.690 | 1.000 |
| 18 | 15 | 5.657 | 0.460 | 10.855 | **0.022** |
| 17 | 16 | -4.682 | -8.687 | -0.677 | **0.009** |
| 18 | 16 | 0.794 | -2.772 | 4.360 | 0.999 |
| 18 | 17 | 5.476 | 1.983 | 8.968 | **<0.001** |
