# Supplementary material for: High uptake of sympagic organic matter by benthos on an Arctic outflow shelf
Source: PLoS One. 2024 Aug 7;19(8):e0308562. doi: 10.1371/journal.pone.0308562 (PMC11305566; doi:10.1371/journal.pone.0308562)
Supplement: S3 Table — (DOCX) [file pone.0308562.s004.docx]

**S3 Table.** Results of a one-way ANOVA comparing means of proportion of sympagic carbon assimilated between stations in benthic invertebrates from northeast Greenland.

|  | **d.f** | **Sum Sq.** | **Mean Sq.** | **F-value** | **P-value** |
| --- | --- | --- | --- | --- | --- |
| *Between stations* | 8 | 12698 | 1587 | 60.02 | **<0.001** |
| *Within stations* | 272 | 7166 | 26 |  |  |
| *Total* | 281 | 19864 |  |  |  |
