# Supplementary material for: High uptake of sympagic organic matter by benthos on an Arctic outflow shelf
Source: PLoS One. 2024 Aug 7;19(8):e0308562. doi: 10.1371/journal.pone.0308562 (PMC11305566; doi:10.1371/journal.pone.0308562)
Supplement: S4 Table — (DOCX) [file pone.0308562.s005.docx]

**S4 Table**. Results of a post-hoc Tukey HSD comparing the means of proportion of sympagic carbon assimilated between stations. Columns *i* and *j* show the stations being compared, while Difference shows the difference in means between those stations. Lower and upper CI are the 95% confidence intervals for the estimated difference in means.

| **Station mean compared** | | **Difference** |  |  |  |
| --- | --- | --- | --- | --- | --- |
| ***i*** | ***j*** | ***i-j*** | **Lower CI** | **Upper CI** | **p-value** |
| 3 | 2 | 21.453 | 17.769 | 25.136 | **<0.001** |
| 5 | 2 | 17.868 | 14.117 | 21.620 | **<0.001** |
| 9 | 2 | 18.918 | 14.856 | 22.980 | **<0.001** |
| 14 | 2 | 8.119 | 3.722 | 12.516 | **<0.001** |
| 15 | 2 | 15.719 | 10.314 | 21.125 | **<0.001** |
| 16 | 2 | 10.736 | 6.948 | 14.523 | **<0.001** |
| 17 | 2 | 15.509 | 11.792 | 19.225 | **<0.001** |
| 18 | 2 | 9.920 | 6.703 | 13.137 | **<0.001** |
| 5 | 3 | -3.584 | -7.604 | 0.436 | 0.124 |
| 9 | 3 | -2.534 | -6.846 | 1.777 | 0.657 |
| 14 | 3 | -13.334 | -17.962 | -8.706 | **<0.001** |
| 15 | 3 | -5.733 | -11.328 | -0.138 | **0.040** |
| 16 | 3 | -10.717 | -14.771 | -6.663 | **<0.001** |
| 17 | 3 | -5.944 | -9.931 | -1.957 | **<0.001** |
| 18 | 3 | -11.533 | -15.059 | -8.006 | **<0.001** |
| 9 | 5 | 1.050 | -3.320 | 5.419 | 0.998 |
| 14 | 5 | -9.750 | -14.432 | -5.067 | **<0.001** |
| 15 | 5 | -2.149 | -7.789 | 3.491 | 0.958 |
| 16 | 5 | -7.133 | -11.249 | -3.017 | **<0.001** |
| 17 | 5 | -2.360 | -6.410 | 1.690 | 0.668 |
| 18 | 5 | -7.949 | -11.546 | -4.351 | **<0.001** |
| 14 | 9 | -10.799 | -15.734 | -5.864 | **<0.001** |
| 15 | 9 | -3.199 | -9.050 | 2.653 | 0.740 |
| 16 | 9 | -8.182 | -12.584 | -3.781 | **<0.001** |
| 17 | 9 | -3.409 | -7.749 | 0.930 | 0.258 |
| 18 | 9 | -8.998 | -12.919 | -5.077 | **<0.001** |
| 15 | 14 | 7.600 | 1.512 | 13.689 | **0.004** |
| 16 | 14 | 2.617 | -2.095 | 7.329 | 0.723 |
| 17 | 14 | 7.390 | 2.736 | 12.044 | **<0.001** |
| 18 | 14 | 1.801 | -2.466 | 6.068 | 0.925 |
| 16 | 15 | -4.984 | -10.648 | 0.681 | 0.135 |
| 17 | 15 | -0.211 | -5.827 | 5.406 | 1.000 |
| 18 | 15 | -5.799 | -11.099 | -0.499 | **0.020** |
| 17 | 16 | 4.773 | 0.689 | 8.857 | **0.009** |
| 18 | 16 | -0.816 | -4.452 | 2.820 | 0.999 |
| 18 | 17 | -5.589 | -9.150 | -2.028 | **<0.001** |
